# Supplementary material for: A causal framework for the drivers of animal social network structure
Source: PLoS Comput Biol. 2025 Sep 8;21(9):e1013370. doi: 10.1371/journal.pcbi.1013370 (PMC12459825; doi:10.1371/journal.pcbi.1013370)
Supplement: S1 Text — Complementary figures, complete definitions of all statistical models, prior predictive simulations, posterior diagnostics, variations on simulation studies 2–4, and alternative parameterisations of several models. (PDF) [file pcbi.1013370.s001.pdf]

Supplementary Materials

A causal framework for the drivers of animal social network structure

Ben Kawam[†], Julia Ostner, Richard McElreath, Oliver Schülke[‡], Daniel Redhead[‡]

[1] Department of Behavioural Ecology, University of Göttingen, Kellnerweg 6, 37077 Göttingen, Germany.
[2] Primate Social Evolution Group, German Primate Center Göttingen, Kellnerweg 4, 37077 Göttingen, Germany.
[3] Department of Human Behaviour, Ecology and Culture, Max Planck Institute for Evolutionary Anthropology, Deutscher Platz 6, 04103 Leipzig, Germany.
[4] Leibniz ScienceCampus Primate Cognition, German Primate Center, Leibniz Institute for Primate Research, Kellnerweg 4, 37077 Göttingen.
[5] Department of Sociology, University of Groningen, Grote Rozenstraat 31, 9712 TG Groningen, The Netherlands.
[6] Inter-University Center for Social Science Theory and Methodology, University of Groningen, Groningen, The Netherlands.
[†]Corresponding author: *Ben Kawam*, bkawam@dpz.eu.
[‡] These authors contributed equally to this work.

Table of Contents

A Simulation study 1: random structuring features 3

B Simulation study 2: individual-level features 8

C Simulation Study 2': categorical individual-level features 11

D Simulation study 3: dyad-level features 15

E Simulation study 3: alternative parameterisation 17

F Simulation Study 3': categorical dyad-level features 19

G Simulation Study 4: kinship in female macaques 24

H Empirical Study 30

I Simulation Study 4': group-level effects 36

J Empirical Study' 40

References 41

A. Simulation study 1: random structuring features

A.1. Observed interactions (y) simulated with SCM 1

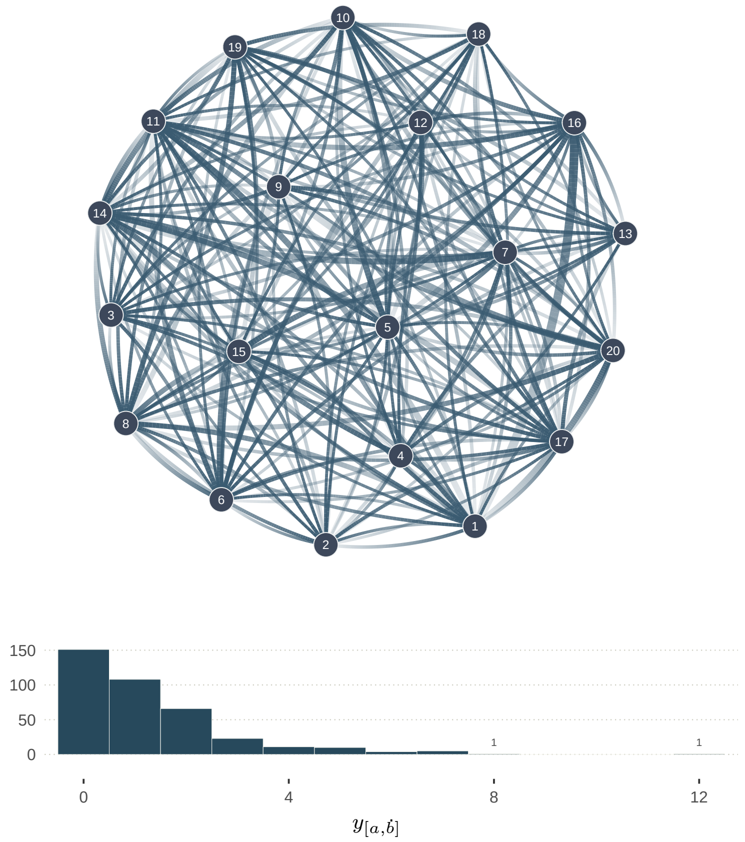

Figure A – *Network of observed interactions (y) generated with SCM 1.* The graph shows 20 individuals (nodes), and the number of observed interactions $y_{[a,b]}$ among them, where a and b correspond to individual indices: $a, b \in \{1, \ldots, 20\}$ and $a \neq b$. The width of the edges indicates the number of observed interactions: 1 interaction for the thinnest, and 12 interactions for the thickest (see distribution under the graph). No edges imply no observed interactions. The transparency gradient of the edges corresponds to the direction of the interaction ($y_{[a,b]}$ or $y_{[b,a]}$): the white end of an edge shows the giver, and its darker end shows the receiver. This network corresponds to the third level of abstraction, in Figure 1.

A.2. Full description of statistical model 1

Below, we write the full statistical model of simulation study 1, as we have implemented it in *Stan*. It differs from the less detailed equations $1.2.1 - 1.2.4$ (see main text) in three aspects. First, each dyad has two directions (from a to b, and from b to a), which simultaneously run in the same Markov Chain. Second, the Multivariate Normal adaptive priors are parameterised as Cholesky factors, for computational reasons. This parameterisation is mathematically equivalent to—but, in this context, more efficient than—the centred parameterisation shown in equations $1.2.3 - 1.2.4$ (see McElreath, 2020; Nicenboim et al., 2021). Third, we show the (here, weakly regularising) priors and hyper-priors. These differences also apply to the other statistical models described in the manuscript.

$$y_{[a,b]} \sim \text{Poisson}(m_{[a,b]})$$
$$y_{[b,a]} \sim \text{Poisson}(m_{[b,a]})$$

$$m_{[a,b]} = \exp(D + G_{[a]} + R_{[b]} + T_{[a,b]})$$
$$m_{[b,a]} = \exp(D + G_{[b]} + R_{[a]} + T_{[b,a]})$$

$$\begin{pmatrix} G_{[a]} \\ R_{[a]} \end{pmatrix} = \begin{pmatrix} s_G & 0 \\ 0 & s_R \end{pmatrix} \times L_{\text{ind}} \times \begin{pmatrix} z_{G_{[a]}} \\ z_{R_{[a]}} \end{pmatrix}$$
$$\begin{pmatrix} T_{[a,b]} \\ T_{[b,a]} \end{pmatrix} = \begin{pmatrix} s_T & 0 \\ 0 & s_T \end{pmatrix} \times L_{\text{dyad}} \times \begin{pmatrix} z_{T_{[a,b]}} \\ z_{T_{[b,a]}} \end{pmatrix}$$

$$D, z_{G_{[a]}}, z_{R_{[a]}}, z_{T_{[a,b]}}, z_{T_{[b,a]}} \sim \text{Normal}(0, 1)$$
$$s_G, s_R, s_T \sim \text{Exponential}(1)$$
$$L_{\text{ind}}, L_{\text{dyad}} \sim \text{LKJ Cholesky}(2).$$

A.3. Prior distributions (simulation study 1)

Using R, we drew n samples for each population-level parameter, or fixed effect (Figure B):

$$\begin{aligned} s_G^{(n)}, s_R^{(n)}, s_T^{(n)} &\sim \text{Exponential}(1) \qquad\qquad n \in \{1, \ldots, 10000\} \\ c_{GR}^{(n)}, c_{TT}^{(n)} &\sim \text{LKJ}(\eta = 2, 2) \\ D^{(n)} &\sim \text{Normal}(0, 1) \end{aligned}$$

Where the *sub*scripts distinguish the parameters from one another (they are not indices), and the *super*script (n) is an index for the prior draws. For instance, $s_G^{(1)}$ is the first draw for the parameter s_G, $s_G^{(2)}$ is the second, and so on. We executed more in depth prior predictive checks for statistical model 4 (see section G.3).

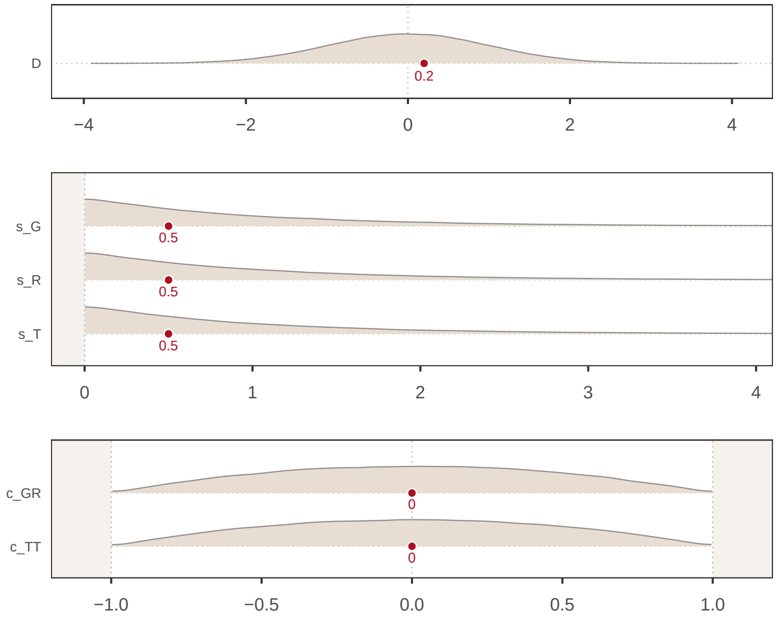

Figure B – *Prior distributions for the fixed effects of statistical model 1.* The prior probability density of a range of parameter values (*x-axis*) is described for several parameters (*y-axis*) of statistical model 1. As for Figure 3, the red dots represent the target values, derived from SCM 1.

A.4. Posterior distributions of m (simulation study 1)

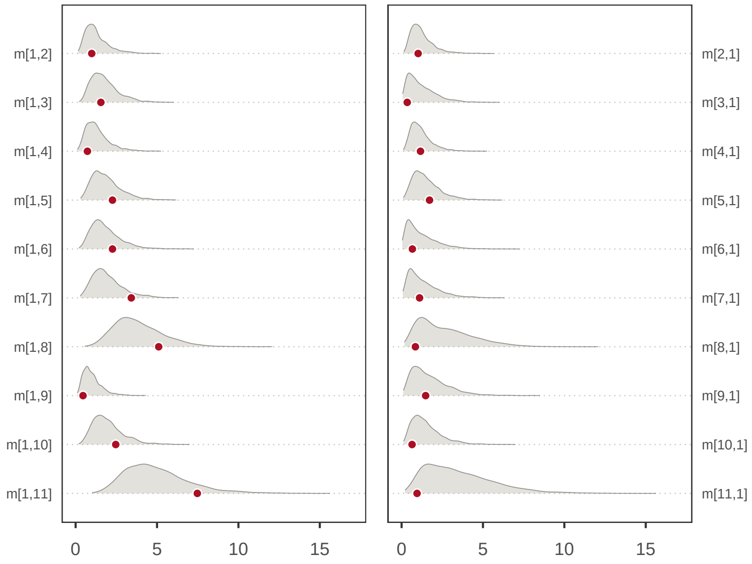

Figure C – *Marginal posterior distributions for a subset of true rates m (posterior model 1).* The posterior probability density of a range of parameter values (x-axis) is described for 20 parameters $m_{[a,b]}$ (y-axis). These parameters correspond to the third level of abstraction of Figure 1. The panel on the left shows the parameters in one direction (a-to-b), and the panel on the right shows them in the other direction (b-to-a). The posterior densities were obtained by updating statistical model 1, with the data generated in SCM 1. The red dots represent the target values, as they are encoded in the SCM.

A.5. Joint distribution for individual-level parameters (simulation study 1)

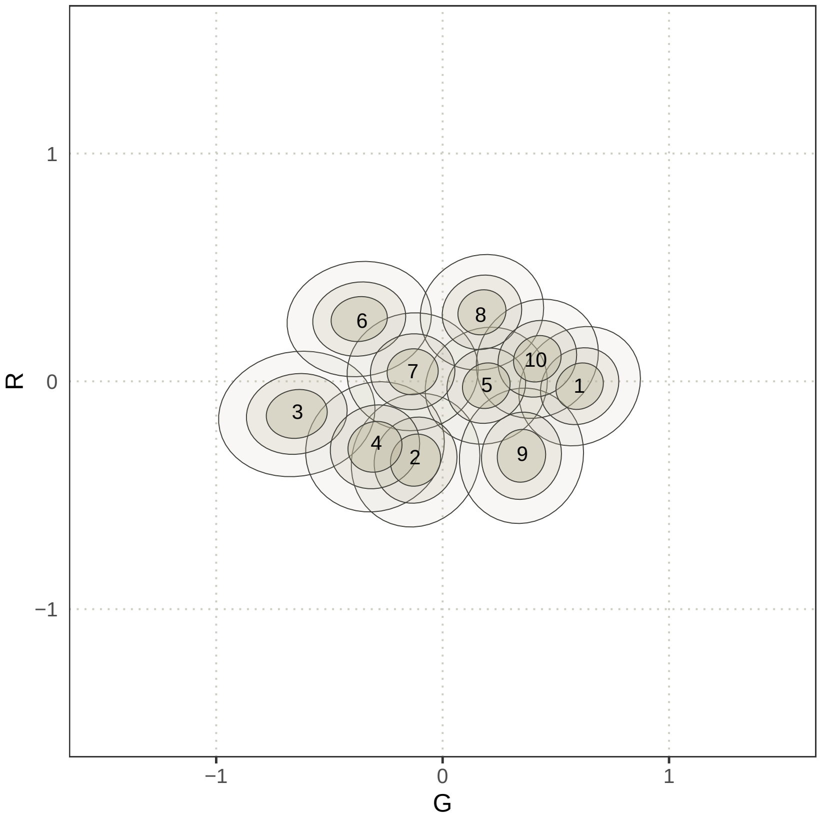

Figure D – *Joint posterior distributions of a subset of individual-level parameters G and R (posterior model 1).* The ellipses approximate 50%, 35%, and 10% percentile intervals, for the posterior probability of joint individual-level parameters (10 individuals shown). A darker colour and a narrower ellipse indicate a higher probability density. Individual indices are shown near the mode of the ellipses. The variances in $G_{[a]}$ and $R_{[a]}$ are respectively estimated by the s_G and s_R parameters (Figure 2c). c_{GR} further quantifies the (lack of) correlation between $G_{[a]}$ and $R_{[a]}$. Figure 3a shows the posterior distributions of $G_{[a]}$ and $R_{[a]}$, by *marginalising* (*i.e.* averaging) over the other parameters.

B. Simulation study 2: individual-level features

B.1. Observed interactions (y) simulated with SCM 2

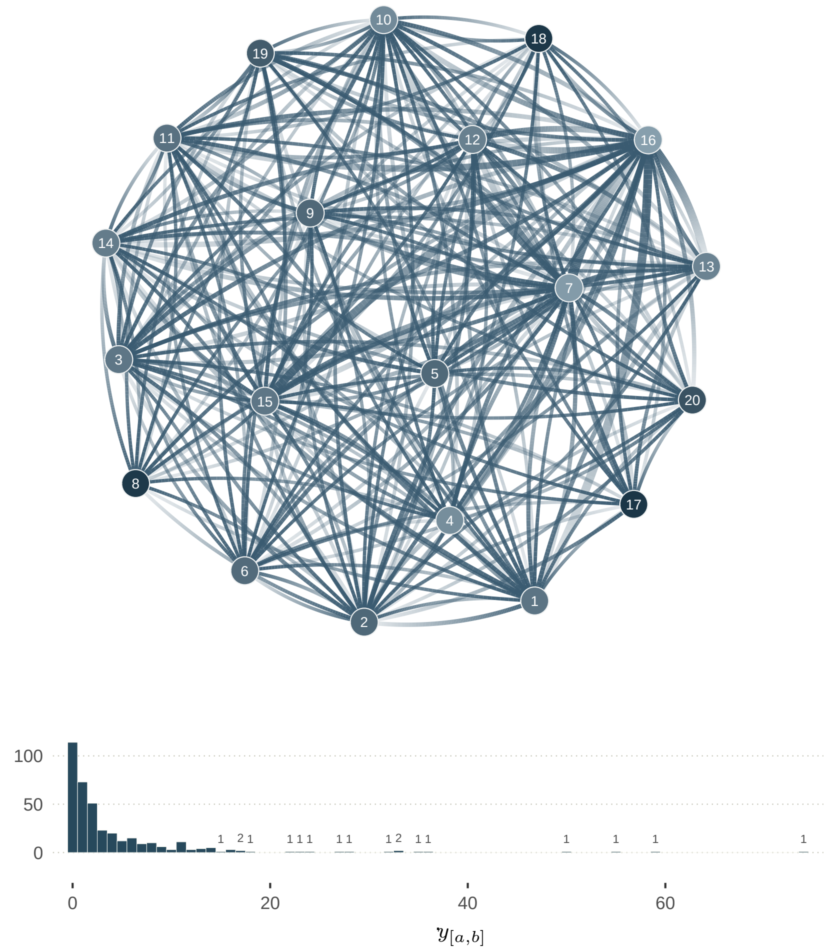

Figure E – *Network of observed interactions (y) generated with SCM 2.* The graph shows 20 individuals (nodes), and the number of observed interactions $y_{[a,b]}$ among them, where a and b correspond to individual indices: $a, b \in \{1, \ldots, 20\}$ and $a \neq b$. The node colour shows the value of $X_{[a]}$: light blue for lower values (*e.g.*, younger individuals), and darker for higher values (*e.g.*, older individuals). The width of the edges indicates the number of observed interactions (see distribution under the graph), where no edges imply no observed interactions. The transparency gradient of the edges corresponds to the direction of the interaction ($y_{[a,b]}$ or $y_{[b,a]}$): the white end of an edge shows the giver, and its darker end shows the receiver. This network corresponds to the third level of abstraction, in Figure 1.

B.2. Full description of statistical model 2

$$y_{[a,b]} \sim \text{Poisson}(m_{[a,b]})$$
$$y_{[b,a]} \sim \text{Poisson}(m_{[b,a]})$$

$$m_{[a,b]} = \exp(D + \hat{G}_{[a]} + \hat{R}_{[b]} + T_{[a,b]})$$
$$m_{[b,a]} = \exp(D + \hat{G}_{[b]} + \hat{R}_{[a]} + T_{[b,a]})$$

$$\hat{G}_{[a]} = G_{[a]} + b_G \cdot X_{[a]}$$
$$\hat{G}_{[b]} = G_{[b]} + b_G \cdot X_{[b]}$$

$$\hat{R}_{[a]} = R_{[a]} + b_R \cdot X_{[a]}$$
$$\hat{R}_{[b]} = R_{[b]} + b_R \cdot X_{[b]}$$

$$\begin{pmatrix} G_{[a]} \\ R_{[a]} \end{pmatrix} = \begin{pmatrix} s_G & 0 \\ 0 & s_R \end{pmatrix} \times L_{\text{ind}} \times \begin{pmatrix} z_{G_{[a]}} \\ z_{R_{[a]}} \end{pmatrix}$$
$$\begin{pmatrix} T_{[a,b]} \\ T_{[b,a]} \end{pmatrix} = \begin{pmatrix} s_T & 0 \\ 0 & s_T \end{pmatrix} \times L_{\text{dyad}} \times \begin{pmatrix} z_{T_{[a,b]}} \\ z_{T_{[b,a]}} \end{pmatrix}$$

$$D, b_G, b_R, z_{G_{[a]}}, z_{R_{[a]}}, z_{T_{[a,b]}}, z_{T_{[b,a]}} \sim \text{Normal}(0, 1)$$
$$s_G, s_R, s_T \sim \text{Exponential}(1)$$
$$L_{\text{ind}}, L_{\text{dyad}} \sim \text{LKJ Cholesky}(2)$$

B.3. Joint distribution for individual-level parameters (simulation study 2)

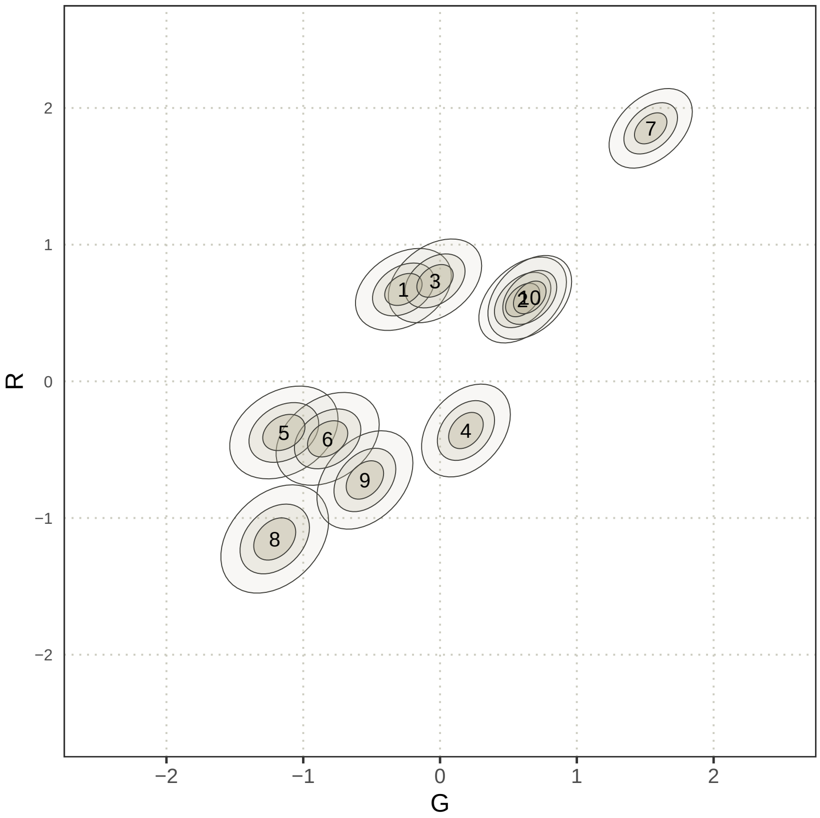

Figure F – *Joint posterior distributions of a subset of individual-level parameters G and R (posterior model 2).* The ellipses approximate 50%, 35%, and 10% percentile intervals, for the posterior probability of individual-level parameters (10 individuals shown). A darker colour and a narrower ellipse indicate a higher probability density. Individual indices are shown near the mode of the ellipses. Note that this plot shows the distribution described by s_G, s_R and c_{GR} (see Figure 4c).

C. Simulation Study 2': categorical individual-level features

This simulation study is a variation of simulation study 2. Its graphical causal model is identical (see Figure 4a-b). However, its quantitative implementation differs. Below, we encode $X_{[a]}$ as categorical variable with two levels, *e.g.* sex, in a SCM (section C.1-C.2), and show that a well-specified estimator (section C.3) can recover its structural parameters (section C.4).

C.1. Structural Causal Model 2'

$$
\begin{aligned}
f_X: &\quad X_{[a]} \in_R \{1, 2\} \\
\\
f_\gamma: &\quad \gamma_{[a]} \sim \begin{cases} \text{Normal}(-0.5, 0.5) & \text{if } X_{[a]} = 1 \\ \text{Normal}(+0.5, 0.5) & \text{if } X_{[a]} = 2 \end{cases} \\
f_\rho: &\quad \rho_{[a]} \sim \begin{cases} \text{Normal}(-0.5, 0.5) & \text{if } X_{[a]} = 1 \\ \text{Normal}(+0.5, 0.5) & \text{if } X_{[a]} = 2 \end{cases} \\
f_\tau: &\quad \tau_{[a,b]} \sim \text{Normal}(0, 0.5) \\
\\
f_m: &\quad m_{[a,b]} = \exp(0.2 + \gamma_{[a]} + \rho_{[b]} + \tau_{[a,b]}) \\
f_y: &\quad y_{[a,b]} \sim \text{Poisson}(m_{[a,b]}).
\end{aligned}
$$

Here, f_X states that the value of X for each individual is random.

C.2. Observed interactions (y) simulated with from SCM 2'

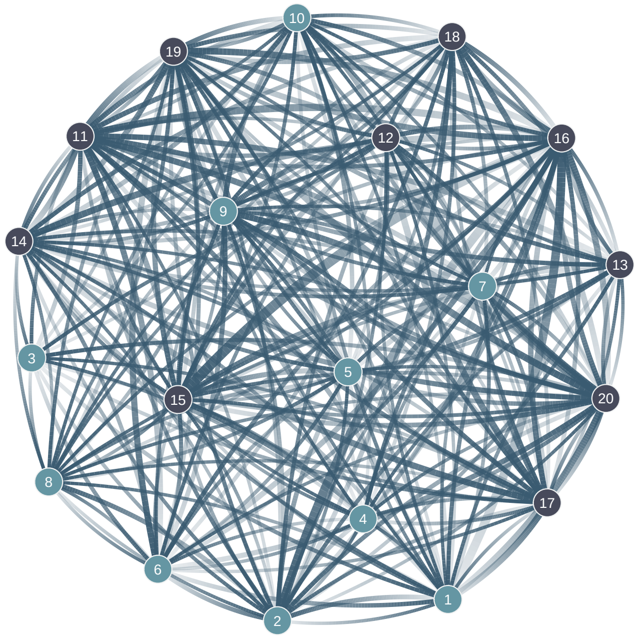

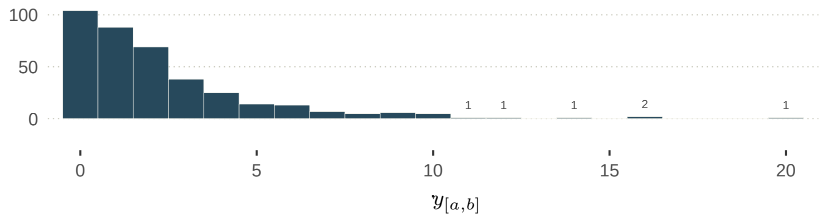

FIGURE G – *Network of observed interactions (y) generated with SCM 2'.* The graph shows 20 individuals (nodes), and the number of observed interactions $y_{[a,b]}$ among them, where a and b correspond to individual indices: $a, b \in \{1, \ldots, 20\}$ and $a \neq b$. The node colour shows the value of $X_{[a]}$: light blue for $X_{[a]} = 1$, and dark blue for $X_{[a]} = 2$. The width of the edges indicates the number of observed interactions (see distribution under the graph) where no edges imply no observed interactions. The transparency gradient of the edges corresponds to the direction of the interaction ($y_{[a,b]}$ or $y_{[b,a]}$): the white end of an edge shows the giver, and its darker end shows the receiver. This network corresponds to the third level of abstraction, in Figure 1.

C.3. Statistical Model 2'

$$y_{[a,b]} \sim \text{Poisson}(m_{[a,b]})$$
$$y_{[b,a]} \sim \text{Poisson}(m_{[b,a]})$$

$$m_{[a,b]} = \exp(D + \hat{G}_{[a]} + \hat{R}_{[b]} + T_{[a,b]})$$
$$m_{[b,a]} = \exp(D + \hat{G}_{[b]} + \hat{R}_{[a]} + T_{[b,a]})$$

$$\hat{G}_{[a]} = L_{[X_{[a]}]} + G_{[a]}$$
$$\hat{G}_{[b]} = L_{[X_{[b]}]} + G_{[b]}$$

$$\hat{R}_{[a]} = M_{[X_{[a]}]} + R_{[a]}$$
$$\hat{R}_{[b]} = M_{[X_{[b]}]} + R_{[b]}$$

$$\begin{pmatrix} G_{[a]} \\ R_{[a]} \end{pmatrix} = \begin{pmatrix} s_G & 0 \\ 0 & s_R \end{pmatrix} \times \mathcal{L}_{\text{ind}} \times \begin{pmatrix} z_{G_{[a]}} \\ z_{R_{[a]}} \end{pmatrix}$$
$$\begin{pmatrix} T_{[a,b]} \\ T_{[b,a]} \end{pmatrix} = \begin{pmatrix} s_T & 0 \\ 0 & s_T \end{pmatrix} \times \mathcal{L}_{\text{dyad}} \times \begin{pmatrix} z_{T_{[a,b]}} \\ z_{T_{[b,a]}} \end{pmatrix}$$

$$D, L_{[1]}, L_{[2]}, M_{[1]}, M_{[2]}, z_{G_{[a]}}, z_{R_{[a]}}, z_{T_{[a,b]}}, z_{T_{[b,a]}} \sim \text{Normal}(0, 1)$$
$$s_G, s_R, s_T \sim \text{Exponential}(1)$$
$$\mathcal{L}_{\text{ind}}, \mathcal{L}_{\text{dyad}} \sim \text{LKJ Cholesky}(2)$$

The nested-brackets notation of $L_{[X_{[a]}]}$ and $M_{[X_{[a]}]}$ indicates that M and L have as many levels as the number of levels in X (*i.e.* here, two levels each), which are indexed based on the value of X for individual a. For instance, if $X_{[a]} = 1$, then $L_{[X_{[a]}]}$ captures the average deviation from D for individuals with $X = 1$; and inversely, if $X_{[b]} = 2$, then $L_{[X_{[b]}]}$ estimates the average deviation from D for individuals with $X = 2$.

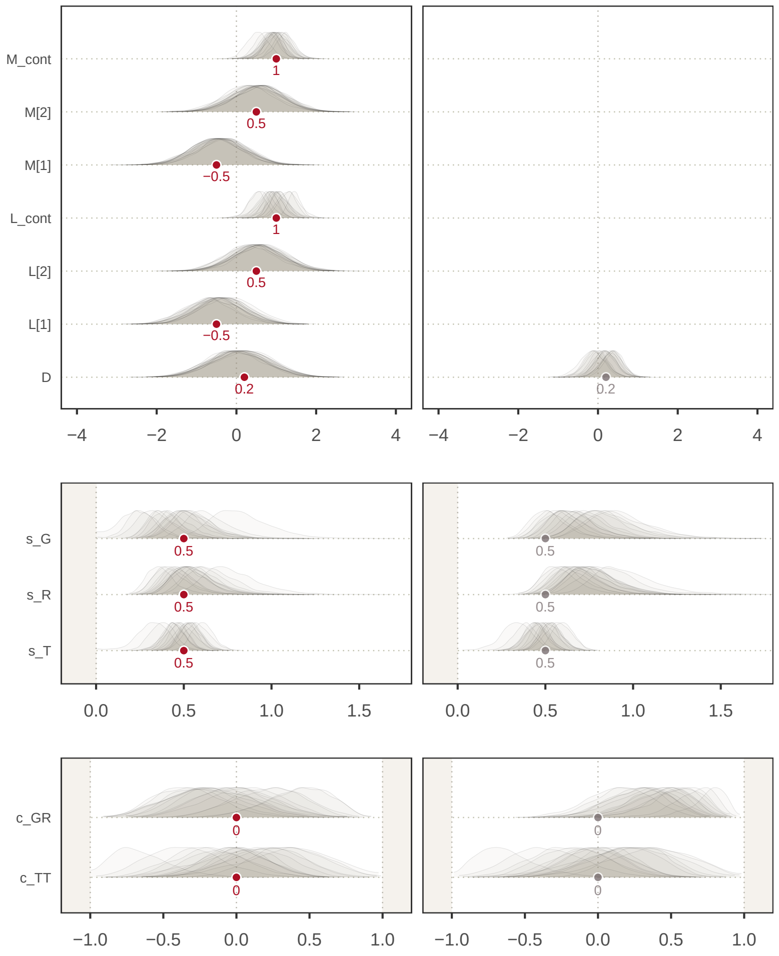

Figure H – *Marginal posterior distributions of the fixed effects of two different statistical models fitted to the data generated with SCM 2'.* Left: fixed effects of the social relations model adjusted by $X_{[a]}$ (see section C.3). Right: fixed effects of the non-adjusted social relations model. "M_cont" and "L_cont" corresponds to the contrasts between $M_{[2]} - M_{[1]}$, and $L_{[2]} - L_{[1]}$, respectively.

D. Simulation study 3: dyad-level features

D.1. Observed interactions (y) simulated with SCM 3

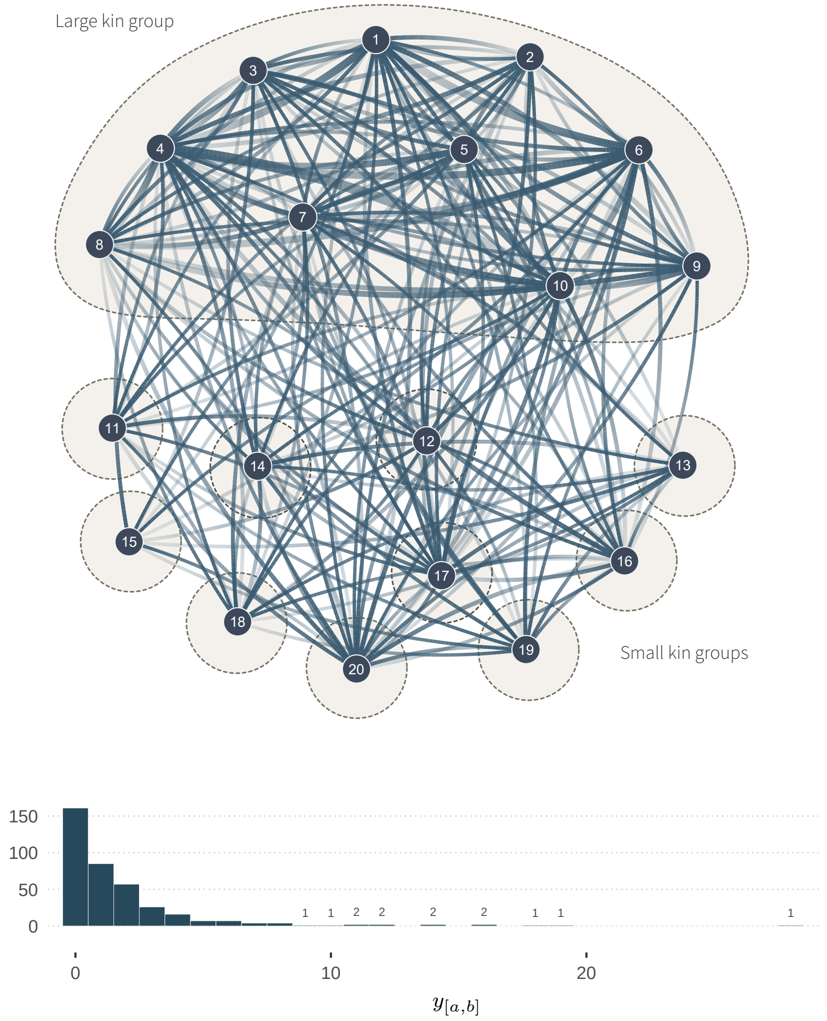

Figure I – *Network of observed interactions (y) generated with SCM 3.* The graph shows 20 individuals (nodes), and the number of observed interactions $y_{[a,b]}$ among them, where a and b correspond to individual indices: $a, b \in \{1, \ldots, 20\}$ and $a \neq b$. The width of the edges indicates the number of observed interactions (see distribution under the graph) where no edges imply no observed interactions. The transparency gradient of the edges corresponds to the direction of the interaction ($y_{[a,b]}$ or $y_{[b,a]}$): the white end of an edge shows the giver, and its darker end shows the receiver. Kin groups are highlighted by dashed outlines. This network corresponds to the third level of abstraction, in Figure 1.

D.2. Full description of statistical model 3

$$y_{[a,b]} \sim \text{Poisson}(m_{[a,b]})$$
$$y_{[b,a]} \sim \text{Poisson}(m_{[b,a]})$$

$$m_{[a,b]} = \exp(D + G_{[a]} + R_{[b]} + \hat{T}_{[a,b]})$$
$$m_{[b,a]} = \exp(D + G_{[b]} + R_{[a]} + \hat{T}_{[b,a]})$$

$$\hat{T}_{[a,b]} = T_{[a,b]} + b_T \cdot X_{|a,b|} + \log(S_{|a,b|})$$
$$\hat{T}_{[b,a]} = T_{[b,a]} + b_T \cdot X_{|b,a|} + \log(S_{|a,b|})$$

$$\begin{pmatrix} G_{[a]} \\ R_{[a]} \end{pmatrix} = \begin{pmatrix} s_G & 0 \\ 0 & s_R \end{pmatrix} \times L_{\text{ind}} \times \begin{pmatrix} z_{G_{[a]}} \\ z_{R_{[a]}} \end{pmatrix}$$
$$\begin{pmatrix} T_{[a,b]} \\ T_{[b,a]} \end{pmatrix} = \begin{pmatrix} s_T & 0 \\ 0 & s_T \end{pmatrix} \times L_{\text{dyad}} \times \begin{pmatrix} z_{T_{[a,b]}} \\ z_{T_{[b,a]}} \end{pmatrix}$$

$$D \sim \text{Normal}(-1, 1)$$
$$b_T, z_{G_{[a]}}, z_{R_{[a]}}, z_{T_{[a,b]}}, z_{T_{[b,a]}} \sim \text{Normal}(0, 1)$$
$$s_G, s_R, s_T \sim \text{Exponential}(1)$$
$$L_{\text{ind}}, L_{\text{dyad}} \sim \text{LKJ Cholesky}(2)$$

E. Simulation study 3: alternative parameterisation

In this section, we show an alternative parameterisation for the DAG, SCM, and statistical model of simulation study 3, where we do not explicitly define γ, ρ, and τ. Instead, we encode the causes of $m_{[a,b]}$ and $y_{[a,b]}$ with arrows pointing directly into them. Here, $Re_{|a,b|}$ affects $m_{[a,b]}$, and $S_{|a,b|}$ affects $y_{[a,b]}$. As a result, $m_{[a,b]}$ corresponds to the true rate of interactions from a to b per *one* time unit. This contrasts with the main text (equations 3.1.6-3.1.7), where $m_{[a,b]}$ defines the rate of interactions per $S_{|a,b|}$ time units. The synthetic data generated from the SCM, and the fixed-effect estimates obtained from the statistical model are identical to those in the main text. This new specification, where the biological variable affects the true rate m, and the sampling effort affects the observed interactions y, might be easier to connect to the three levels of abstraction of Figure 1. It would be a good choice if we were solely interested in deriving an adjustment set from the DAG and estimating a causal effect. In the main text, we use the parameterisation with γ, ρ, and τ, for it made causal assumptions very explicit, and it allowed us to show how individual- and dyad-level structuring features could affect $G_{[a]}$, $R_{[b]}$, and $T_{[a,b]}$ (Figure 5b).

E.1. Directed Acyclic Graph

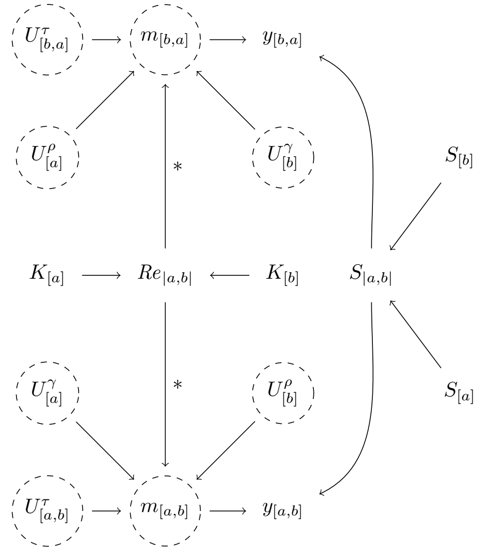

Figure J – *Alternative DAG for simulation study 3.*

Alternative parameterisation of SCM 3

$K_{[a]}$ is assigned to individuals following the description in the main text—*i.e.*, there are 10 individuals in one large kin group, and the other individuals are alone in their kin group.

$$
\begin{aligned}
f_{Re}: &\quad Re_{|a,b|} = \begin{cases} 1 & \text{if } K_{[a]} = K_{[b]} \\ 0 & \text{if } K_{[a]} \neq K_{[b]} \end{cases} \\
f_{S_{[a]}}: &\quad S_{[a]} \sim \text{Uniform}(1, 2.5) \\
f_{S_{|a,b|}}: &\quad S_{|a,b|} = S_{[a]} + S_{[b]} \\
\\
f_{U^{\gamma}}: &\quad U^{\gamma}_{[a]} \sim \text{Normal}(0, 0.5) \\
f_{U^{\rho}}: &\quad U^{\rho}_{[a]} \sim \text{Normal}(0, 0.5) \\
f_{U^{\tau}}: &\quad U^{\tau}_{[a,b]} \sim \text{Normal}(0, 0.5) \\
\\
f_{m}: &\quad m_{[a,b]} \sim \exp(-1.2 + U^{\gamma}_{[a]} + U^{\rho}_{[b]} + U^{\tau}_{[a,b]} + 0.8^{*} \cdot \tilde{Re}_{|a,b|}) \\
f_{y}: &\quad y_{[a,b]} \sim \text{Poisson}(m_{[a,b]} \cdot S_{|a,b|})
\end{aligned}
$$

Alternative parameterisation of statistical model 3

$$
\begin{aligned}
y_{[a,b]} &\sim \text{Poisson}(m_{[a,b]} \cdot S_{|a,b|}) \\
y_{[b,a]} &\sim \text{Poisson}(m_{[b,a]} \cdot S_{|a,b|})
\end{aligned}
$$

$$
\begin{aligned}
m_{[a,b]} &= \exp(D + G_{[a]} + R_{[b]} + T_{[a,b]} + b^{*}_{T} \cdot \tilde{Re}_{|a,b|}) \\
m_{[b,a]} &= \exp(D + G_{[b]} + R_{[a]} + T_{[b,a]} + b^{*}_{T} \cdot \tilde{Re}_{|a,b|})
\end{aligned}
$$

$$
\begin{aligned}
\begin{pmatrix} G_{[a]} \\ R_{[a]} \end{pmatrix} &= \begin{pmatrix} s_G & 0 \\ 0 & s_R \end{pmatrix} \times L_{\text{ind}} \times \begin{pmatrix} z_{G_{[a]}} \\ z_{R_{[a]}} \end{pmatrix} \\
\begin{pmatrix} T_{[a,b]} \\ T_{[b,a]} \end{pmatrix} &= \begin{pmatrix} s_T & 0 \\ 0 & s_T \end{pmatrix} \times L_{\text{dyad}} \times \begin{pmatrix} z_{T_{[a,b]}} \\ z_{T_{[b,a]}} \end{pmatrix}
\end{aligned}
$$

$$
\begin{gathered}
D \sim \text{Normal}(-1, 1) \\
b^{*}_{T}, z_{G_{[a]}}, z_{R_{[a]}}, z_{T_{[a,b]}}, z_{T_{[b,a]}} \sim \text{Normal}(0, 1) \\
s_G, s_R, s_T \sim \text{Exponential}(1) \\
L_{\text{ind}}, L_{\text{dyad}} \sim \text{LKJ Cholesky}(2)
\end{gathered}
$$

F. Simulation Study 3': categorical dyad-level features

This simulation study is a variation of simulation study 3. Its causal structure (Figure K) is identical to Figure 5a-b. However, its quantitative implementation differs. Here, the dyadic variable $X_{[a,b]}$ (equivalent of $Re_{|a,b|}$ in simulation 3) is a categorical variable, *e.g.* the combination of sexes for a given directed dyad. The effect of $X_{[a,b]}$ is parameterised as a two-by-two matrix, encoding an effect for each combination of sexes (section F.2-F.3). We show that a well-specified estimator (section F.4) can recover the SCM's structural parameters (section F.5).

F.1. Directed Acyclic Graph 3'

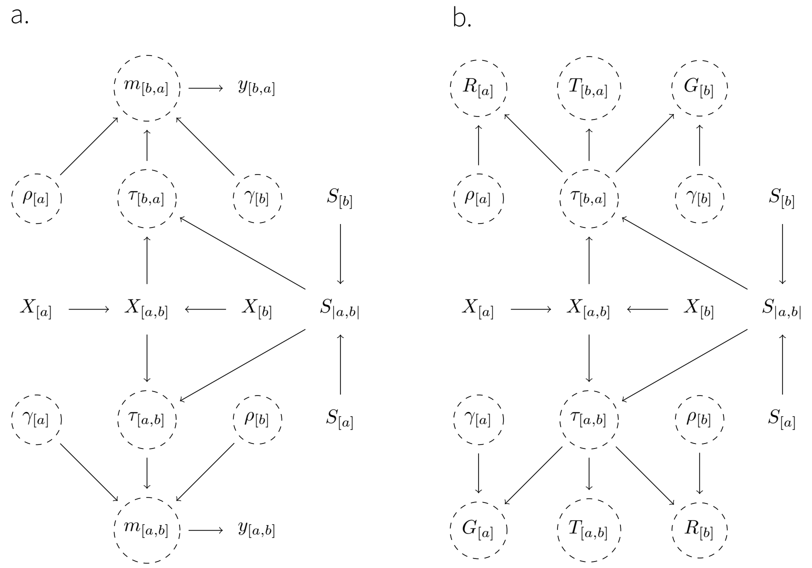

Figure K – *Directed Acyclic Graph for simulation study 3'.* $X_{[a]}$ represents the sex of an individual, a, and $X_{[a,b]}$, the combination of sexes for a given directed dyad. Males are encoded as 1, and females as 2. For example, if a is a male and b is female, then $X_{[a,b]} = X_{[1,2]}$. S represents the sampling effort for each individual ($S_{[a]}$) and dyad ($S_{|a,b|}$).

F.2. Structural Causal Model 3'

$$
\begin{aligned}
f_{S_{[a]}}: &\quad S_{[a]} \sim \text{Uniform}(1, 2.5) \\
f_{S_{|a,b|}}: &\quad S_{|a,b|} = S_{[a]} + S_{[b]} \\
f_{X_{[a]}}: &\quad X_{[a]} \in_R \{1, 2\} \\
f_{X_{[a,b]}}: &\quad X_{[a,b]} = [X_{[a]}, X_{[b]}] \\
f_{\psi}: &\quad \psi = \begin{pmatrix} -1 & 0 \\ 0.5 & 0.5 \end{pmatrix} \\
\\
f_{\gamma}: &\quad \gamma_{[a]} \sim \text{Normal}(0, 0.5) \\
f_{\rho}: &\quad \rho_{[a]} \sim \text{Normal}(0, 0.5) \\
f_{\tau}: &\quad \tau_{[a,b]} \sim \text{Normal}(\log(S_{|a,b|}) + \psi_{S_{[a,b]}}, 0.5) \\
\\
f_{m}: &\quad m_{[a,b]} = \exp(-1.2 + \gamma_{[a]} + \rho_{[b]} + \tau_{[a,b]}) \\
f_{y}: &\quad y_{[a,b]} \sim \text{Poisson}(m_{[a,b]})
\end{aligned}
$$

F.3. Observed interactions (y) simulated with from SCM 3'

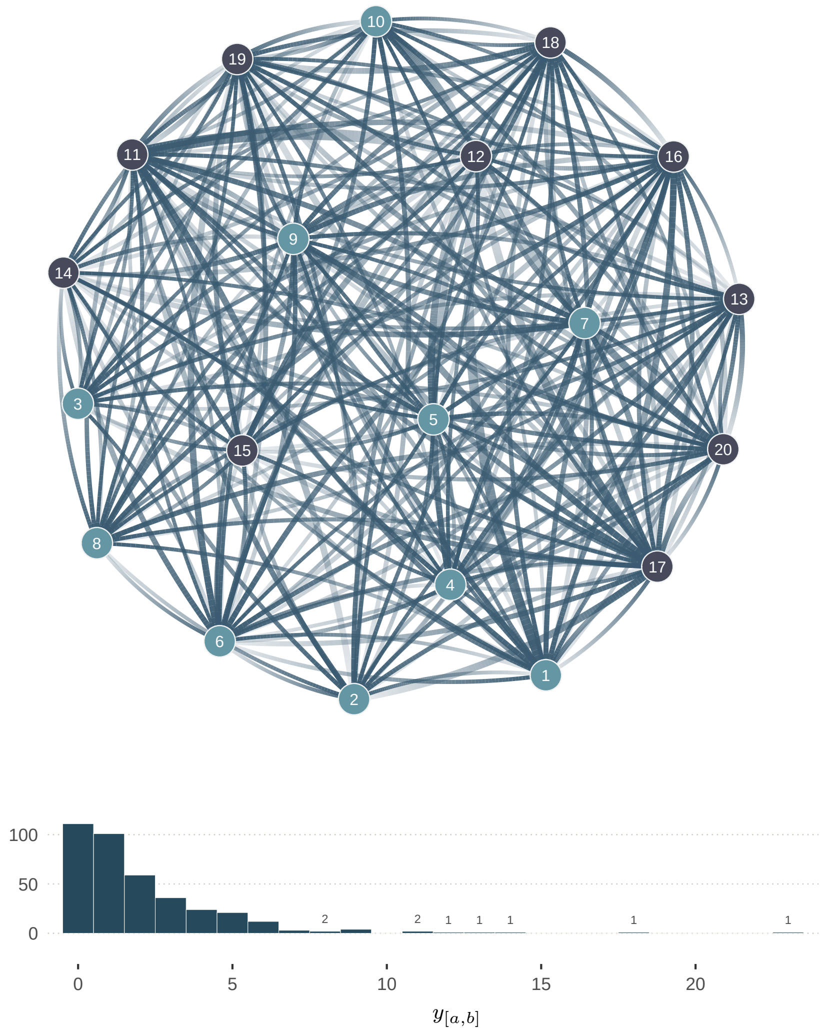

Figure L – *Network of observed interactions (y) generated with SCM 3'.* The graph shows 20 individuals (nodes), and the number of observed interactions $y_{[a,b]}$ among them, where a and b correspond to individual indices: $a, b \in \{1, \dots, 20\}$ and $a \neq b$. The node colour shows the value of $X_{[a]}$: light blue for males, and dark blue for females. The width of the edges indicates the number of observed interactions (see distribution under the graph) where no edges imply no observed interactions. The transparency gradient of the edges corresponds to the direction of the interaction ($y_{[a,b]}$ or $y_{[b,a]}$): the white end of an edge shows the giver (start of the arrow), and its darker end shows the receiver (head of the arrow). This network corresponds to the third level of abstraction, in Figure 1.

F.4. Full description of statistical model 3'

$$y_{[a,b]} \sim \text{Poisson}(m_{[a,b]})$$
$$y_{[b,a]} \sim \text{Poisson}(m_{[b,a]})$$

$$m_{[a,b]} = \exp(D + G_{[a]} + R_{[b]} + \hat{T}_{[a,b]})$$
$$m_{[b,a]} = \exp(D + G_{[b]} + R_{[a]} + \hat{T}_{[b,a]})$$

$$B = \begin{pmatrix} b_{[1,1]} & b_{[1,2]} \\ b_{[2,1]} & b_{[2,2]} \end{pmatrix}$$

$$\hat{T}_{[a,b]} = \log(S_{|a,b|}) + T_{[a,b]} + B_{X_{[a,b]}}$$
$$\hat{T}_{[b,a]} = \log(S_{|a,b|}) + T_{[b,a]} + B_{X_{[b,a]}}$$

$$\begin{pmatrix} G_{[a]} \\ R_{[a]} \end{pmatrix} = \begin{pmatrix} s_G & 0 \\ 0 & s_R \end{pmatrix} \times L_{\text{ind}} \times \begin{pmatrix} z_{G_{[a]}} \\ z_{R_{[a]}} \end{pmatrix}$$
$$\begin{pmatrix} T_{[a,b]} \\ T_{[b,a]} \end{pmatrix} = \begin{pmatrix} s_T & 0 \\ 0 & s_T \end{pmatrix} \times L_{\text{dyad}} \times \begin{pmatrix} z_{T_{[a,b]}} \\ z_{T_{[b,a]}} \end{pmatrix}$$

$$D \sim \text{Normal}(-1, 1)$$
$$b_j, z_{G_{[a]}}, z_{R_{[a]}}, z_{T_{[a,b]}}, z_{T_{[b,a]}} \sim \text{Normal}(0, 1)$$
$$s_G, s_R, s_T \sim \text{Exponential}(1)$$
$$L_{\text{ind}}, L_{\text{dyad}} \sim \text{LKJ Cholesky}(2)$$

F.5. Posterior models (simulation study 3')

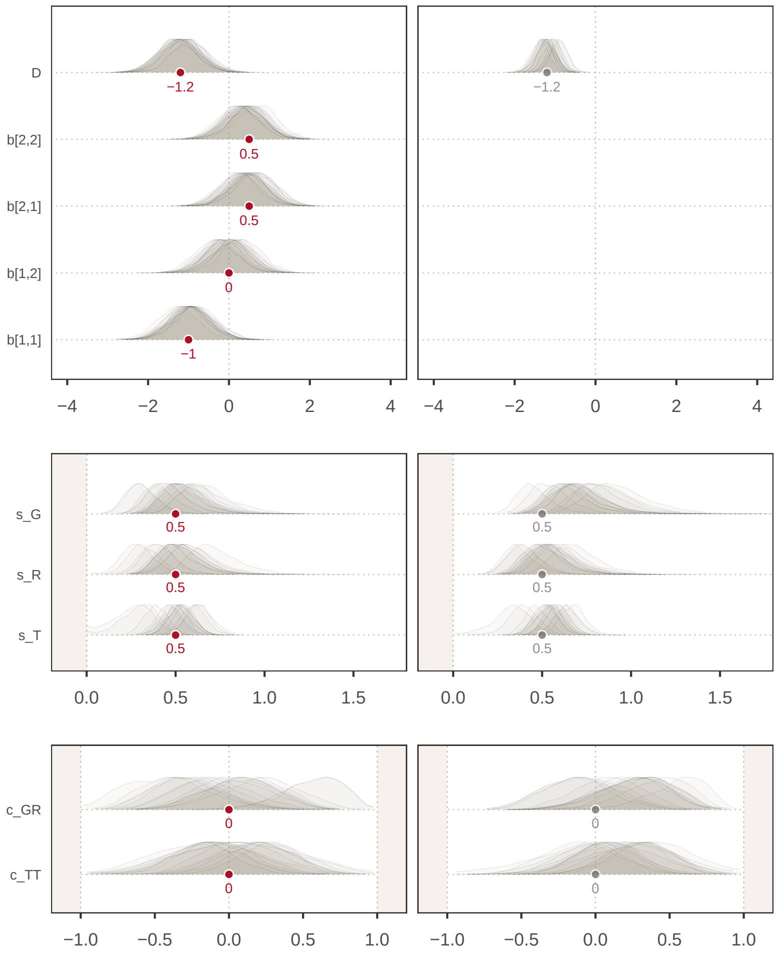

Figure M – *Marginal posterior distributions of the fixed effects of two different statistical models fitted to the data generated with SCM 3'.* Left: fixed effects of the social relations model adjusted by $X_{[a,b]}$ and $S_{|a,b|}$ (see section F.4). Right: fixed effects of the estimator adjusted by $S_{|a,b|}$ only. This second statistical model is identical to the one in section F.4, but without the $B_{X_{[a,b]}}$ parameters.

G. Simulation Study 4: kinship in female macaques

G.1. Observed interactions (y) simulated with SCM 4

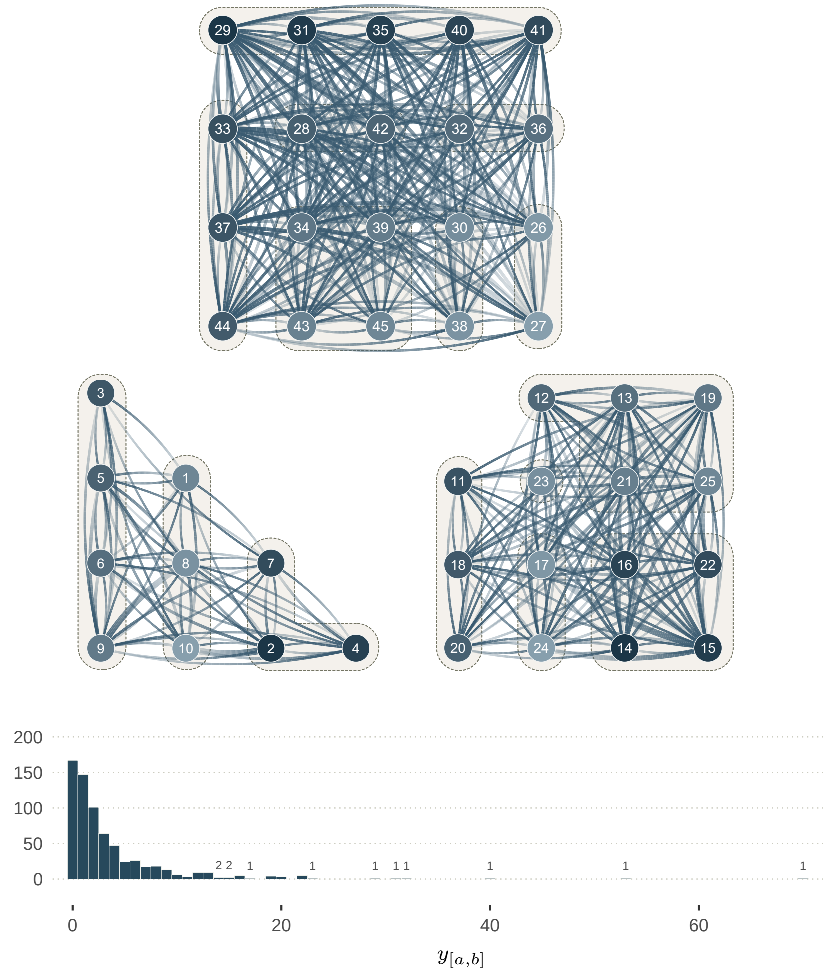

Figure N – *Network of observed interactions (y) generated with SCM 4.* The graph shows 45 individuals (nodes) across three groups of 10, 15, and 20 individuals, respectively. The edges show the interactions $y_{[a,b]}$ among them. Their width indicates the number of observed interactions (see distribution under the graphs) where no edges imply no observed interactions. The transparency gradient of the edges corresponds to the direction of the interaction ($y_{[a,b]}$ or $y_{[b,a]}$): the white end of an edge shows the giver (start of the arrow), and its darker end shows the receiver (head of the arrow). The colour of the node corresponds to the individual dominance rank: lighter for low ranks, and darker for higher ranks. Kin group are highlighted by dashed outlines. This network corresponds to the third level of abstraction, in Figure 1.

G.2. Full description of statistical model 4

$$y_{[a,b]} \sim \text{Poisson}(m_{[a,b]})$$
$$y_{[b,a]} \sim \text{Poisson}(m_{[b,a]})$$

$$m_{[a,b]} = \exp(D + G_{[a]} + R_{[b]} + \hat{T}_{[a,b]})$$
$$m_{[b,a]} = \exp(D + G_{[b]} + R_{[a]} + \hat{T}_{[b,a]})$$

$$\hat{T}_{[a,b]} = T_{[a,b]} + b_{Re} \cdot Re_{|a,b|} + \log(S_{|a,b|}) + \begin{cases} b_{Ra_1} \cdot (Ra_{[b]} - Ra_{[a]}) & \text{if } Ra_{[a]} < Ra_{[b]} \\ b_{Ra_2} \cdot (Ra_{[b]} - Ra_{[a]}) & \text{if } Ra_{[a]} \geq Ra_{[b]} \end{cases}$$

$$\hat{T}_{[b,a]} = T_{[b,a]} + b_{Re} \cdot Re_{|a,b|} + \log(S_{|a,b|}) + \begin{cases} b_{Ra_1} \cdot (Ra_{[a]} - Ra_{[b]}) & \text{if } Ra_{[b]} < Ra_{[a]} \\ b_{Ra_2} \cdot (Ra_{[a]} - Ra_{[b]}) & \text{if } Ra_{[b]} \geq Ra_{[a]} \end{cases}$$

$$\begin{pmatrix} G_{[a]} \\ R_{[a]} \end{pmatrix} = \begin{pmatrix} s_G & 0 \\ 0 & s_R \end{pmatrix} \times L_{\text{ind}} \times \begin{pmatrix} z_{G_{[a]}} \\ z_{R_{[a]}} \end{pmatrix}$$
$$\begin{pmatrix} T_{[a,b]} \\ T_{[b,a]} \end{pmatrix} = \begin{pmatrix} s_T & 0 \\ 0 & s_T \end{pmatrix} \times L_{\text{dyad}} \times \begin{pmatrix} z_{T_{[a,b]}} \\ z_{T_{[b,a]}} \end{pmatrix}$$

$$D \sim \text{Normal}(-1.5, 1)$$
$$z_{G_{[a]}}, z_{R_{[a]}}, z_{T_{[a,b]}}, z_{T_{[b,a]}} \sim \text{Normal}(0, 1)$$
$$b_{Re}, b_{Ra_1}, b_{Ra_2} \sim \text{Normal}(0, 0.6)$$
$$s_G, s_R, s_T \sim \text{Exponential}(1)$$
$$L_{\text{ind}}, L_{\text{dyad}} \sim \text{LKJ Cholesky}(3)$$

G.3. Prior predictive simulations

In this section, we describe the prior predictive checks that we conducted for statistical model 4. Using R, we started by drawing n samples for each of the nine population-level parameters (fixed effects):

$$
\begin{aligned}
s_G^{(n)}, s_R^{(n)}, s_T^{(n)} &\sim \text{Exponential}(1) \qquad\qquad n \in \{1, \ldots, 1000\} \\
c_{GR}^{(n)}, c_{TT}^{(n)} &\sim \text{LKJ}(\eta = 3, 2) \\
b_{Re}^{(n)}, b_{Ra_1}^{(n)}, b_{Ra_2}^{(n)} &\sim \text{Normal}(0, 0.6) \\
D^{(n)} &\sim \text{Normal}(-1.5, 1)
\end{aligned}
$$

Where the *sub*scripts distinguish the parameters from one another, and the *super*script (n) is an index for the prior draws. Using these parameters, we defined two prior variance-covariance matrices Σ: one for the individual-level and one for the dyad-level varying effects.

$$
\begin{aligned}
\Sigma_{\text{ind}}^{(n)} &= \begin{pmatrix} s_G^{(n)} s_G^{(n)} & c_{GR}^{(n)} s_G^{(n)} s_R^{(n)} \\ c_{GR}^{(n)} s_G^{(n)} s_R^{(n)} & s_R^{(n)} s_R^{(n)} \end{pmatrix} \\
\Sigma_{\text{dyad}}^{(n)} &= \begin{pmatrix} s_T^{(n)} s_T^{(n)} & c_{TT}^{(n)} s_T^{(n)} s_T^{(n)} \\ c_{TT}^{(n)} s_T^{(n)} s_T^{(n)} & s_T^{(n)} s_T^{(n)} \end{pmatrix}
\end{aligned}
$$

Next, we constructed the three groups containing, respectively, 10, 15, and 20 individuals (45 individuals in total), resulting in 340 dyads and 680 directed dyads across the three groups. For each prior sample n, we drew 20 individual-level parameters $G_{[a]}$, 20 individual-level parameters $R_{[a]}$, and 680 dyad-level parameters $T_{[a,b]}$ and $T_{[b,a]}$ (340 in each direction).

$$
\begin{aligned}
\begin{pmatrix} G_{[a]}^{(n)} \\ R_{[a]}^{(n)} \end{pmatrix} &\sim \text{MVNormal}\left[\begin{pmatrix} 0 \\ 0 \end{pmatrix}, \Sigma_{\text{ind}}^{(n)}\right] \\
\begin{pmatrix} T_{[a,b]}^{(n)} \\ T_{[b,a]}^{(n)} \end{pmatrix} &\sim \text{MVNormal}\left[\begin{pmatrix} 0 \\ 0 \end{pmatrix}, \Sigma_{\text{dyad}}^{(n)}\right]
\end{aligned}
$$

For each sample n, we also assigned a rank to each individual, and a value of genetic relatedness for each dyad:

$$
\begin{aligned}
Ra_{[a]}^{(n)} &\sim \text{Uniform}(0, 1) \\
Re_{|a,b|}^{(n)} &\in_R \{0, 1\}
\end{aligned}
$$

Finally, we deterministically obtained an expected rate $m_{[a,b]}^{(n)}$, per directed dyad and

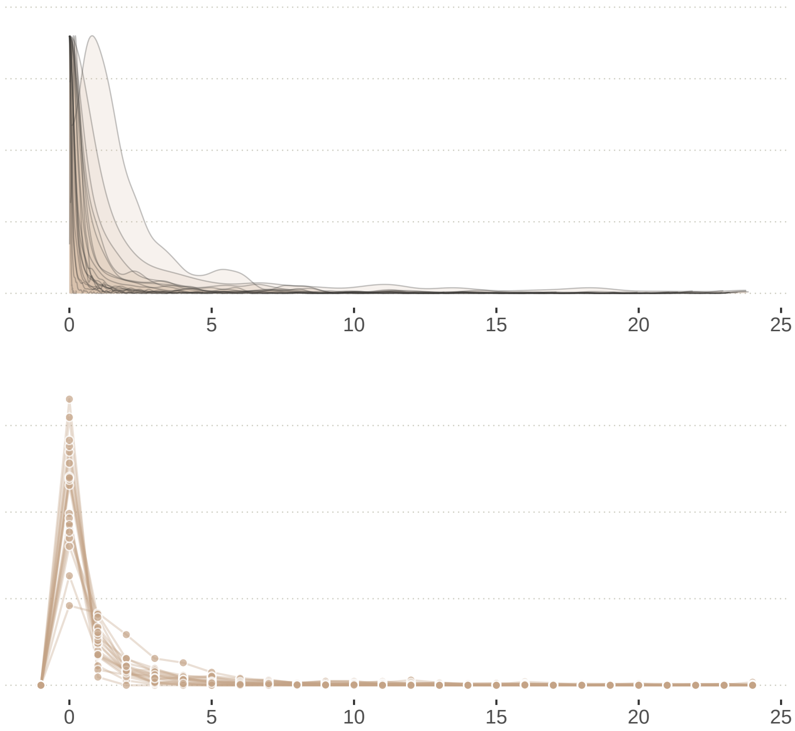

Figure O – ***Top:*** *Distribution of prior average interaction rates* $m^{(n)}_{[a,b]}$. Each slab represents the distribution of 680 directed rates, for one prior draw n. We show the distribution for 20 prior samples n, *i.e.* 20 slabs. ***Bottom:*** *Distribution of prior predictions* $y^{(n)}_{[a,b]}$. Each sequence of dots connected by one line represent the counts of observed interactions $y_{[a,b]}$ across 680 directed dyads, for one prior sample n. Again, we show 20 distributions, one for each prior sample n.

per prior sample.

$$\hat{T}^{(n)}_{[a,b]} = T^{(n)}_{[a,b]} + b^{(n)}_{Re} \cdot Re^{(n)}_{|a,b|} + \begin{cases} b^{(n)}_{Ra_1} \cdot (Ra^{(n)}_{[b]} - Ra^{(n)}_{[a]}) & \text{if } R^{(n)}_{[a]} < R^{(n)}_{[b]} \\ b^{(n)}_{Ra_1} \cdot (Ra^{(n)}_{[b]} - Ra^{(n)}_{[a]}) & \text{if } R^{(n)}_{[a]} \geq R^{(n)}_{[b]} \end{cases}$$

$$m^{(n)}_{[a,b]} = \exp(D^{(n)} + G^{(n)}_{[a]} + R^{(n)}_{[b]} + \hat{T}^{(n)}_{[a,b]})$$

We show the distribution of $m^{(n)}_{[a,b]}$ and $m^{(n)}_{[b,a]}$ for 20 (out of 1000) prior draws in Figure O (top panel). From this rate, we drew a prior prediction y per directed dyad and per prior sample (Figure O, bottom panel):

$$y^{(n)}_{[a,b]} \sim \text{Poisson}(m^{(n)}_{[a,b]}).$$

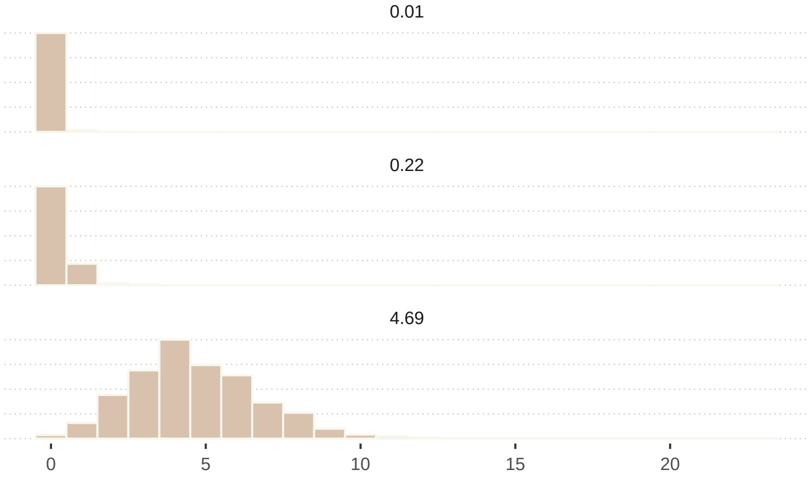

Figure P – Distribution of predicted observations $y_{[a,b]}^{(n)}$ for dyads with low-, medium-, and high-rates $\hat{m}_k$ of interactions.

To better understand what the prior model implies, we summarised the distribution of rates $m_{[a,b]}^{(n)}$ in terms of its 10%, 50%, and 90% percentiles. We call these summary values $\hat{m}_k$. They take the following values: $\hat{m}_1 = 0.01$, $\hat{m}_2 = 0.22$, $\hat{m}_3 = 4.69$. That is, the prior *average* interaction rates per directed dyad roughly ranges from 1 interaction every 100 days, to about 4 or 5 interaction per day. We then drew 1000 prior predictions y for each value $\hat{m}_k$ (Figure P).

$$y_k^{(n)} \sim \text{Poisson}(\hat{m}_k).$$

In summary, our prior model is compatible with a broad range of dyadic grooming interaction rates: dyadic rates of almost zero, as well as rates of several interactions per day, are plausible before the estimator it is updated with data.

To visualise the prior distribution of slopes, we started by computing the variation across directed dyads due to unobserved network structuring features:

$$\psi_{[a,b]}^{(n)} = D^{(n)} + G_{[a]}^{(n)} + R_{[b]}^{(n)} + T_{[a,b]}^{(n)}$$

We selected 24 samples n (out of 1000), and computed an interaction rate $m_{[a,b]}^{(n,j)}$ for the whole range of a predictor variable $X^{(j)}$:

$$m_{[a,b]}^{(n,j)} = \exp(\psi_{[a,b]}^{(n)} + b_{Re}^{(n)} \cdot X^{(j)})$$

Where $X^{(j)}$ is a vector of size 101, ranging from 0 to 1. $X^{(1)} = 0$, $X^{(2)} = 0.01$, $X^{(3)} = 0.02$, and so on, until $X^{(101)} = 1$. X represents either $Re_{|a,b|}$ or $\Delta Ra_{|a,b|}$

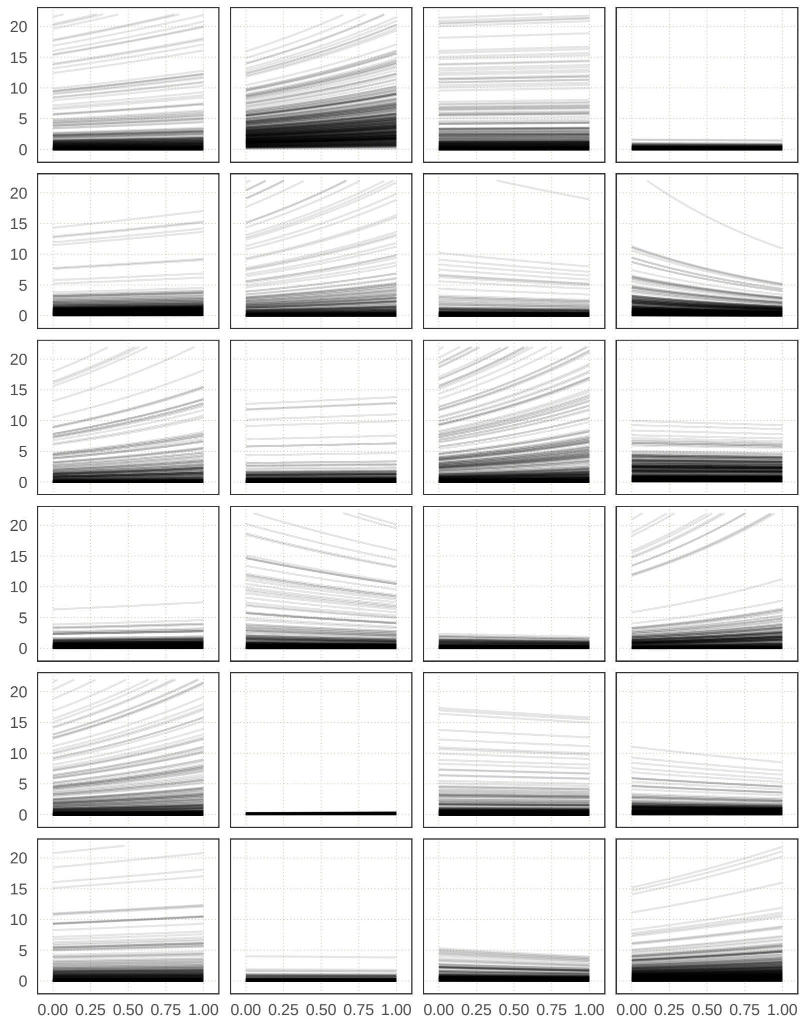

Figure Q – *Distribution of prior slopes for* 24 *prior samples.* The x-axis represents the range of a dyad-level predictor variable (difference in rank or genetic relatedness), the y-axis represents $m_{[a,b]}$. Each panel corresponds to one prior sample n, and contains 680 slopes (one for each directed-dyad).

(in the case of $Re_{|a,b|}$, only the first and last values of the vector apply). Note that $b^{(n)}_{Re}$ is interchangeable with $b^{(n)}_{Ra_1}$ and $b^{(n)}_{Ra_2}$, for they have the same prior distribution. We plot the resulting prior slopes in Figure Q. Within each panel, we can visualise the difference across dyads that exists for a given prior sample n—it is, in this regard, complimentary to Figure O. Across the 20 panels, we observe the variation implied by the prior probability distribution of the slope.

H. Empirical Study

The plots shown in this section either represent the empirical data directly (section H.1), or show the output of models that were updated with the empirical data (sections H.2-H.5).

H.1. Distribution of sampling effort and observed interaction rates

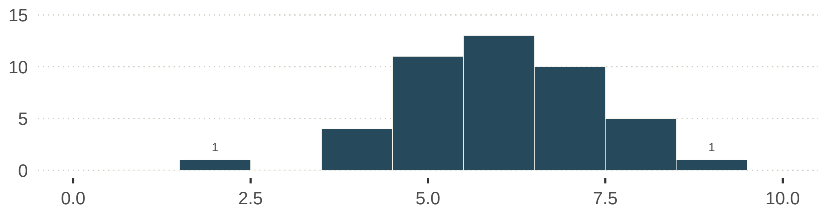

Figure R – *Distribution of sampling effort $S_{[a]}$, in full days, across 45 individuals.* The unit of $S_{[a]}$ is 12 hours. The lowest value of $S_{[a]}$ is 1.82 days (22 hours), and the highest is 9.02 days (108.3 hours).

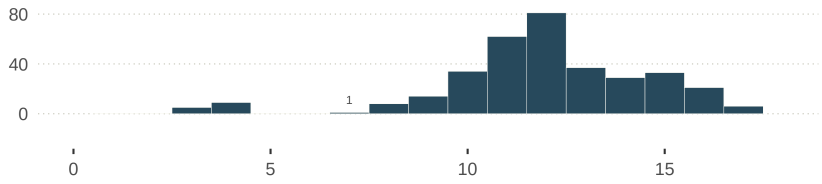

Figure S – *Distribution of sampling effort $S_{|a,b|}$, in full days, across 340 dyads.* The unit of $S_{|a,b|}$ is 12 hours. The lowest value of $S_{|a,b|}$ is 3.1 days (37.6 hours), and the highest is 17.3 days (207.9 hours).

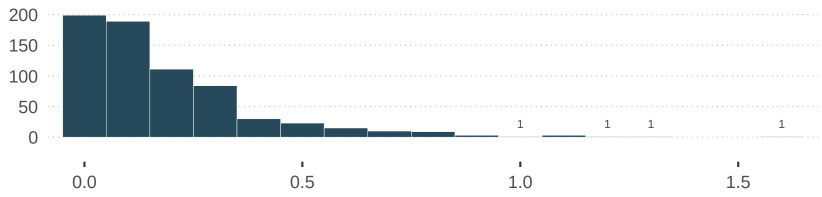

Figure T – *Distribution of observed interaction rates:* $(y_{[a,b]}/S_{|a,b|})$. The unit of $S_{|a,b|}$ is 12 hours.

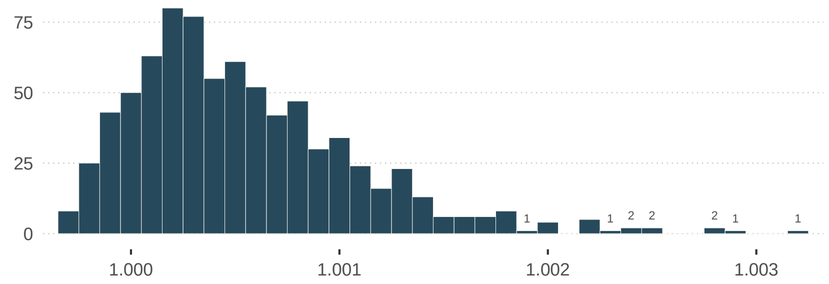

Figure U – *Distribution of $\hat{R}$ across all parameters of statistical model 4 fitted to the empirical data* (Vehtari et al., 2021).

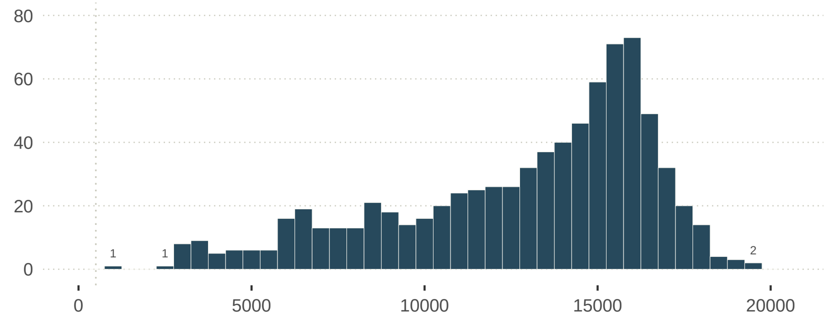

Figure V – *Distribution of the effective number of MCMC samples (bulk), across all parameters of statistical model 4 fitted to the empirical data.* The vertical line marks 500.

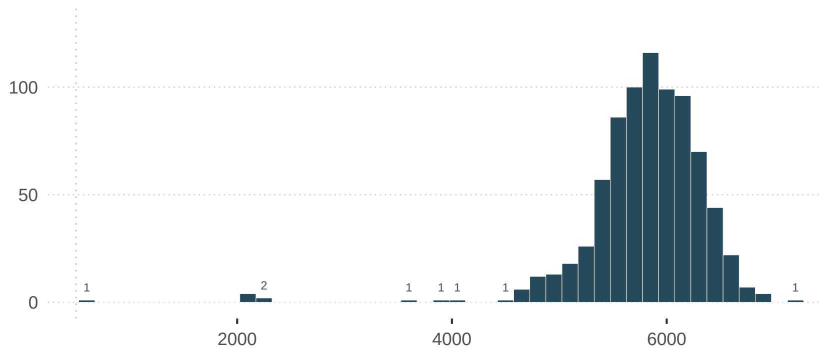

Figure W – *Distribution of the effective number of MCMC samples (tail), across all parameters of statistical model 4 fitted to the empirical data.* The vertical line marks 500.

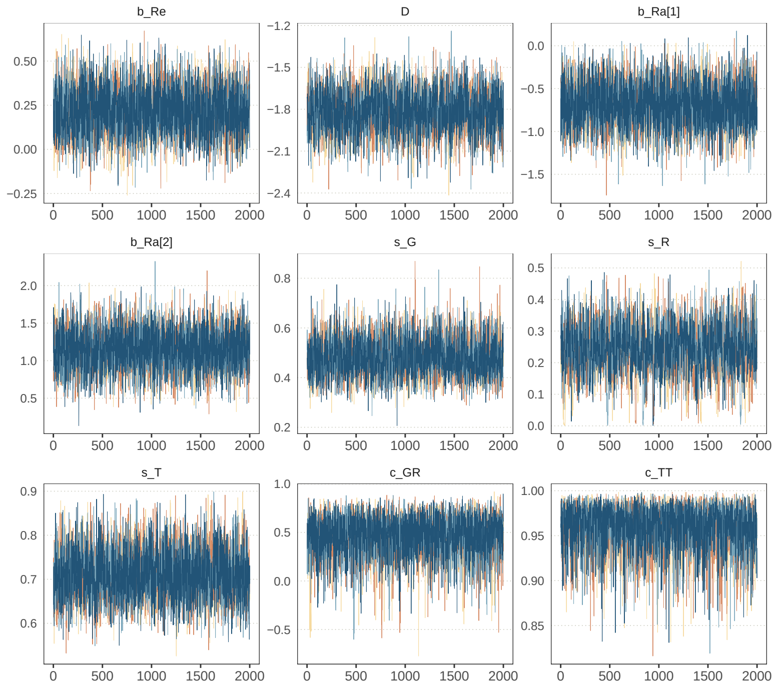

Figure X – *MCMC traceplots for the model's fixed effects.* Each of the four chains (four different colours) was run for 4000 iterations: 2000 warm-up draws, and 2000 post warm-up draws (shown here).

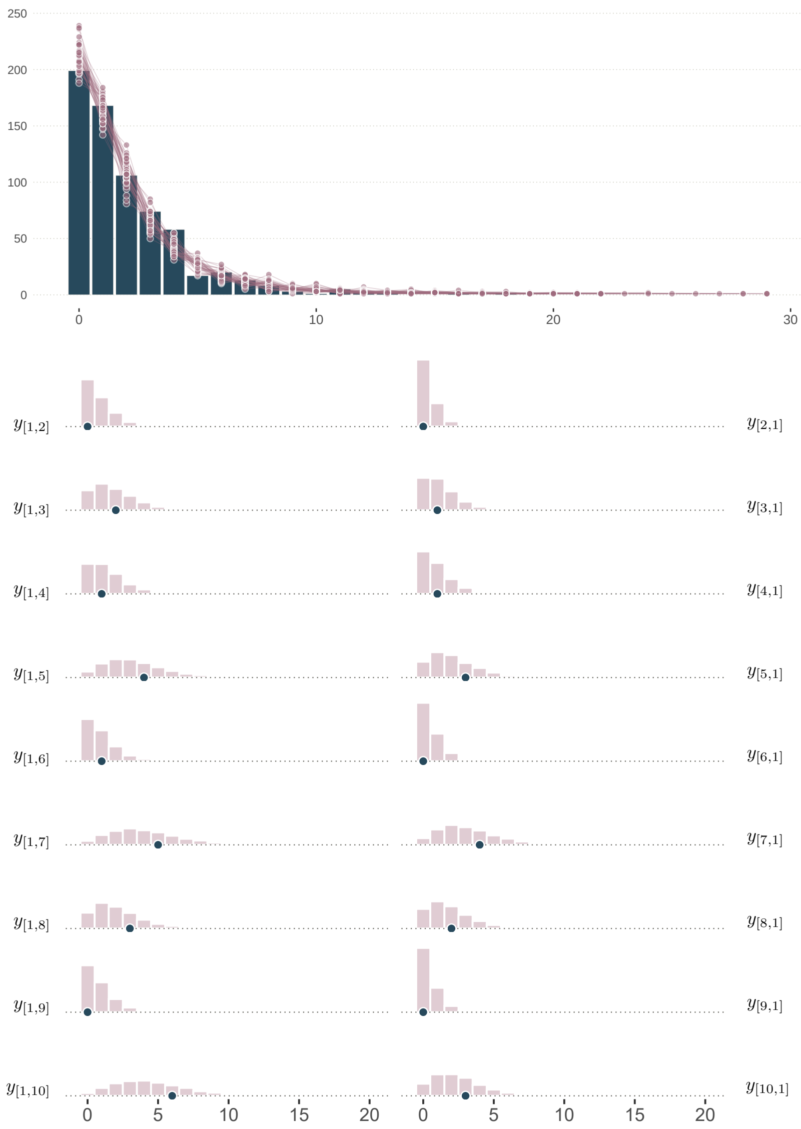

Figure Y – Top: *Posterior predictive distribution of* $y_{[a,b]}$. The empirical distribution is shown in blue (same as in Figure 7c), and the posterior predictive distribution across all dyads is shown in pink. Predictions from the same posterior sample (40 samples in total) are connected by a line. Bottom: *Dyad-specific posterior predictive distribution of* $y_{[a,b]}$*, for 9/340 dyads.* The pink histograms represent posterior predictions for each dyad. The observed values of $y_{[a,b]}$ are shown in blue.

H.4. Posterior distribution of baseline levels $\psi_{[a,b]}$

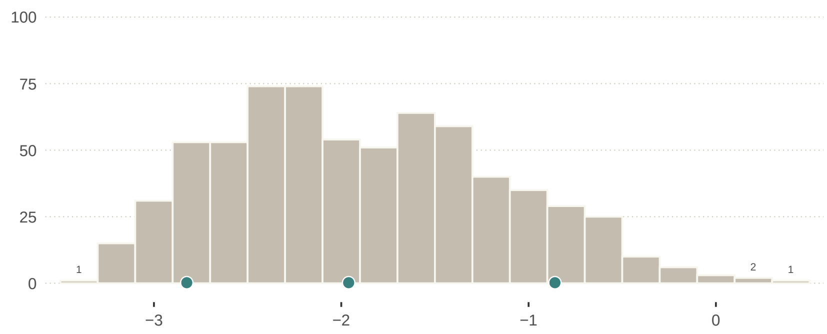

Figure Z – *Distribution of $\psi_{[a,b]}$'s posterior means.* The blue dots indicate, from left to right: ψ_1, ψ_2, and ψ_3.

H.5. Effect of dominance rank

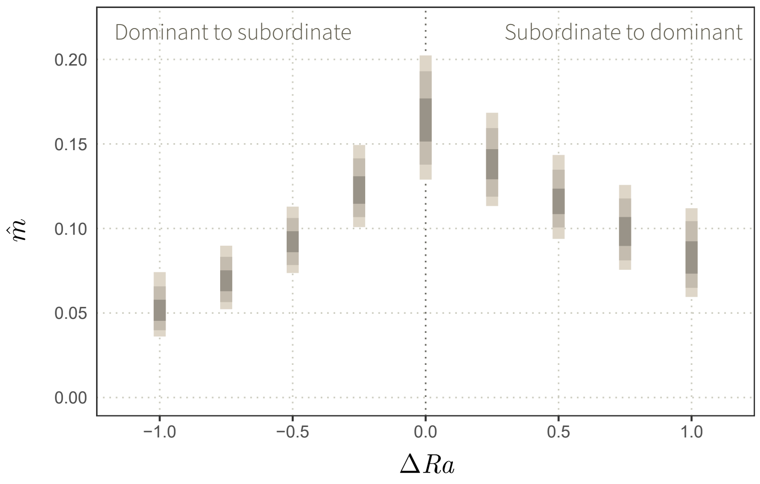

Figure ZA – *Effect of dominance rank on* m*.* Posterior draws of rates $\hat{m}_k$ (bouts/day), for average dyads only varying in ΔRa. The intervals of increasing density respectively contain 89%, 75%, and 40% of posterior samples.

To estimate the effect of dominance rank, we compute nine posterior distributions $\hat{m}_k^{(n)}$ (Figure ZA):

$$\hat{m}_k^{(n)} = D^{(n)} + \begin{cases} b_{Ra_1}^{(n)} \cdot \Delta Ra_k & \text{if } \Delta Ra_k \geq 0 \\ b_{Ra_2}^{(n)} \cdot \Delta Ra_k & \text{if } \Delta Ra_k < 0, \end{cases}$$

Where n corresponds to 1000 posterior samples and ΔRa_k are differences in dominance rank ranging from -1 to 1, for $k \in \{1, \ldots, 9\}$. Note that we slightly abuse notation, here. We also named some parameters $\hat{m}_k$ in the section dedicated to prior predictive simulations (section G.3) but $\hat{m}_k$ refer to a *different* set of parameters here.

How to interpret Figure ZA given the assumptions encoded in the statistical (section G.2) and causal (Figure 6a) models:

$\hat{m}_k$ represents directed interaction rates in nine counterfactual worlds changing only in ΔRa (the other causes of m are constant, and the individuals are assumed to be non-related). Comparing these rates from one another allows us to isolate the causal effect of dominance rank, even if it is generically confounded by relatedness in nature. We see that when an individual faces a higher-ranked individual, its rate of grooming decreases: the bigger the distance in rank, the bigger the decrease (right panel). Likewise, when an individual faces an individual that is lower ranked, its grooming rate decreases as well, such that a larger distance implies a larger decrease (left panel). Together, this suggests that rank causes individuals to groom one another more frequently when they are proximate in the dominance hierarchy.

I. Simulation Study 4': group-level effects

Below, we present an slightly more realistic version of simulation study 4. The new elements of simulation study 4' regard group size $GS_{\text{gr}_{|a,b|}}$. This group-level variable is now assumed to affect interaction rates, such that in smaller groups, dyads tend to have a higher rate of social interactions. We conclude this section by fitting the resulting estimator (statistical model 4') to the empirical dataset.

I.1. Causal Models 4'

$\delta_{\text{gr}_{|a,b|}}$ encodes the causal effect of group-level variables like group size on $m_{[a,b]}$ (Figure ZBa). δ is thus analogous to γ, ρ, and τ, but for group-level effects.

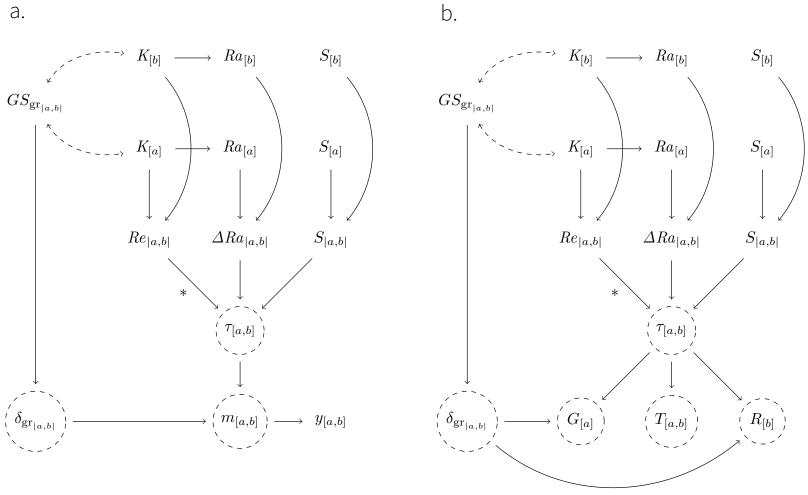

Figure ZB – *Directed Acyclic Graph for simulation study 4'.* The graph is similar to Figure 6a, except for the effect of group size $GS_{\text{gr}_{|a,b|}}$. Here, $GS_{\text{gr}_{|a,b|}}$ determines the value of $\delta_{\text{gr}_{|a,b|}}$, which then affects the rate $m_{[a,b]}$ for all dyads in the group of individuals a and b.

SCM 4' is very similar to SCM 4. The only differences regard the global intercept, and are encoded through f_δ and f_m:

$$f_\delta: \quad \delta_{\text{gr}_{|a,b|}} = \begin{cases} -0.8 \text{ if } GS_{\text{gr}_{|a,b|}} = 10 \\ -1.2 \text{ if } GS_{\text{gr}_{|a,b|}} = 15 \\ -1.6 \text{ if } GS_{\text{gr}_{|a,b|}} = 20 \end{cases}$$

$$f_m: \quad m_{[a,b]} = \exp(\delta_{\text{gr}_{|a,b|}} + \gamma_{[a]} + \rho_{[b]} + \tau_{[a,b]}).$$

I.2. Observed interactions (y) simulated with SCM 4'

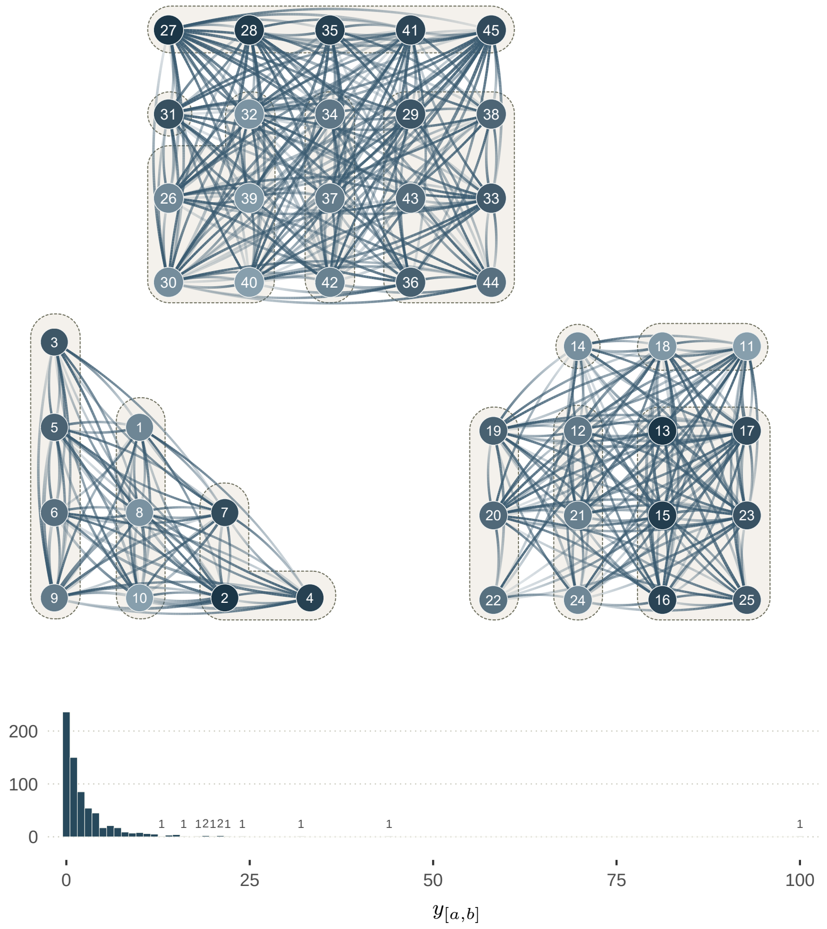

Figure ZC – *Network of observed interactions (y) generated with SCM 4'.* The graph shows 45 individuals (nodes) across three groups of 10, 15, and 20 individuals, respectively. The edges show the interactions $y_{[a,b]}$ among them. Their width indicates the number of observed interactions (see distribution under the graphs) where no edges imply no observed interactions. The transparency gradient of the edges corresponds to the direction of the interaction ($y_{[a,b]}$ or $y_{[b,a]}$): the white end of an edge shows the giver (start of the arrow), and its darker end shows the receiver (head of the arrow). The colour of the node corresponds to the individual dominance rank: lighter for low ranks, and darker for higher ranks. Kin group are highlighted by dashed outlines. This network corresponds to the third level of abstraction, in Figure 1.

I.3. Full description of statistical model 4'

$$y_{[a,b]} \sim \text{Poisson}(m_{[a,b]})$$
$$y_{[b,a]} \sim \text{Poisson}(m_{[b,a]})$$

$$m_{[a,b]} = \exp(d_{[\text{gr}_{|a,b|}]} + G_{[a]} + R_{[b]} + \hat{T}_{[a,b]})$$
$$m_{[b,a]} = \exp(d_{[\text{gr}_{|a,b|}]} + G_{[b]} + R_{[a]} + \hat{T}_{[b,a]})$$

$$\hat{T}_{[a,b]} = T_{[a,b]} + b_{Re} \cdot Re_{|a,b|} + \log(S_{|a,b|}) + \begin{cases} b_{Ra_1} \cdot (Ra_{[b]} - Ra_{[a]}) & \text{if } Ra_{[a]} < Ra_{[b]} \\ b_{Ra_2} \cdot (Ra_{[b]} - Ra_{[a]}) & \text{if } Ra_{[a]} \geq Ra_{[b]} \end{cases}$$
$$\hat{T}_{[b,a]} = T_{[b,a]} + b_{Re} \cdot Re_{|a,b|} + \log(S_{|a,b|}) + \begin{cases} b_{Ra_1} \cdot (Ra_{[a]} - Ra_{[b]}) & \text{if } Ra_{[b]} < Ra_{[a]} \\ b_{Ra_2} \cdot (Ra_{[a]} - Ra_{[b]}) & \text{if } Ra_{[b]} \geq Ra_{[a]} \end{cases}$$

$$\begin{pmatrix} G_{[a]} \\ R_{[a]} \end{pmatrix} = \begin{pmatrix} s_G & 0 \\ 0 & s_R \end{pmatrix} \times L_{\text{ind}} \times \begin{pmatrix} z_{G_{[a]}} \\ z_{R_{[a]}} \end{pmatrix}$$
$$\begin{pmatrix} T_{[a,b]} \\ T_{[b,a]} \end{pmatrix} = \begin{pmatrix} s_T & 0 \\ 0 & s_T \end{pmatrix} \times L_{\text{dyad}} \times \begin{pmatrix} z_{T_{[a,b]}} \\ z_{T_{[b,a]}} \end{pmatrix}$$

$$d_{[1]}, d_{[2]}, d_{[3]} \sim \text{Normal}(-1.5, 1)$$
$$z_{G_{[a]}}, z_{R_{[a]}}, z_{T_{[a,b]}}, z_{T_{[b,a]}} \sim \text{Normal}(0, 1)$$
$$b_{Re}, b_{Ra_1}, b_{Ra_2} \sim \text{Normal}(0, 0.6)$$
$$s_G, s_R, s_T \sim \text{Exponential}(1)$$
$$L_{\text{ind}}, L_{\text{dyad}} \sim \text{LKJ Cholesky}(3)$$

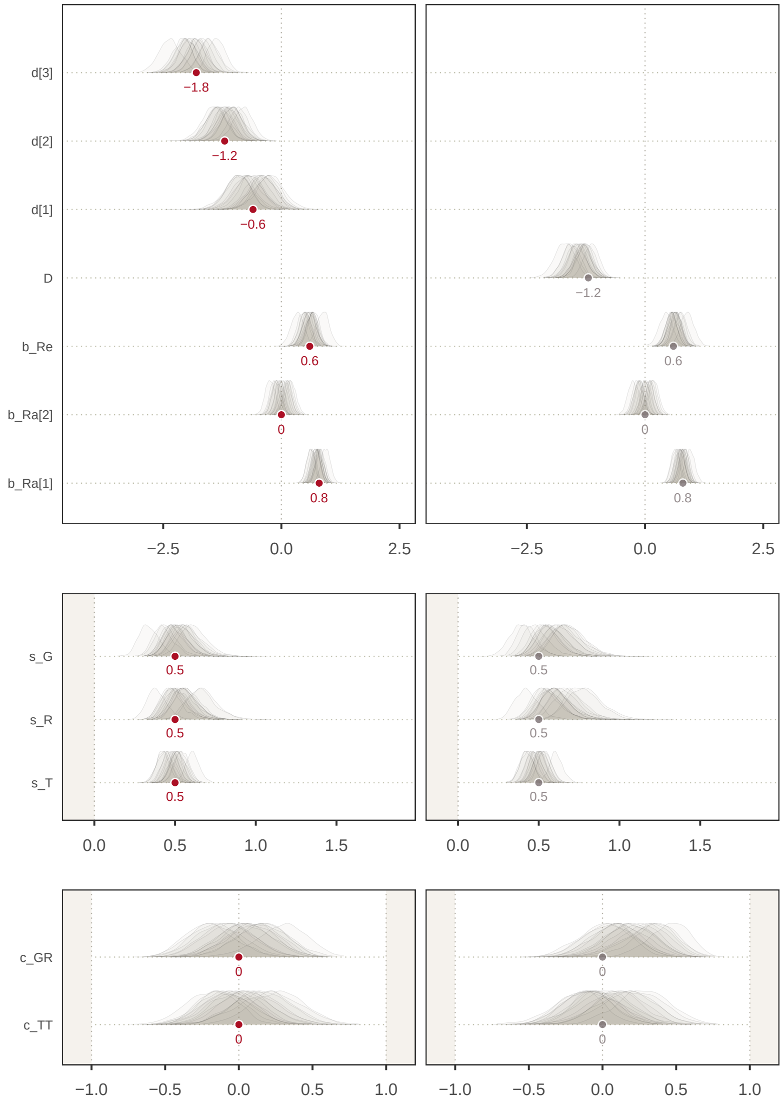

FIGURE ZD – *Simulation study 4'.* **b.** *Left*: fixed effects of the social relations model adjusted by $Re_{|a,b|}$, $S_{|a,b|}$, $\Delta Ra_{|a,b|}$, and group gr (statistical model 4'). *Right*: fixed effects of the social relations model adjusted by $Re_{|a,b|}$, $S_{|a,b|}$, and $\Delta Ra_{|a,b|}$ (statistical model 4). The target values of the fully-adjusted model are shown in grey.

The path that we wish to estimate ($Re_{|a,b|}$ to $m_{[a,b]}$) is confounded by group size $GS_{\text{gr}_{|a,b|}}$. However, an examination of Figure ZD (right panel) tells us that statistical model 4 could recover the true causal effect of $Re_{|a,b|}$, even if it did not include group size. This is because in SCM 4', $GS_{\text{gr}_{|a,b|}}$ causes individuals belonging to larger groups to give, and receive, fewer interactions. These *inter-individual* differences (unit level) are fully captured by G and R (Figures ZBb and ZD, right panel), thereby blocking the biasing effect of group size on the rates m (sub-unit level).

J. Empirical Study'

Here, we show the posterior distributions of statistical model 4' (see sections I.1-I.4), fitted to the empirical data.

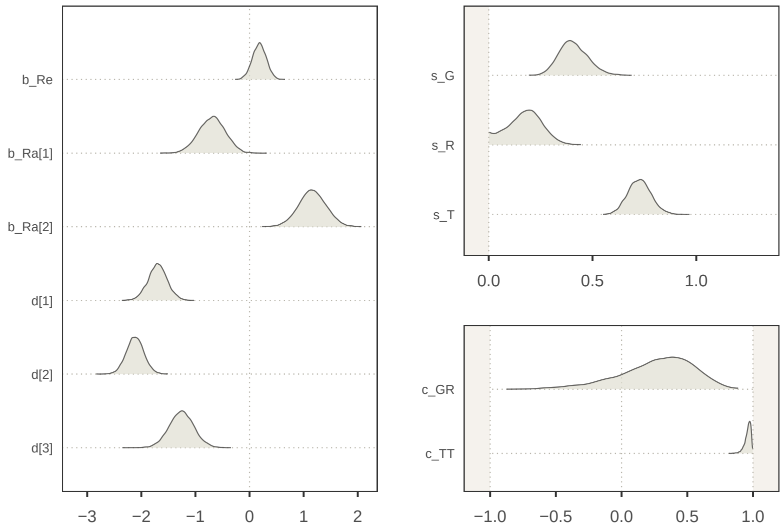

Figure ZE – *Marginal posterior distribution of the fixed effects of statistical model 4', updated with empirical data from female macaques.*

References

McElreath, R. (2020). *Statistical rethinking: A Bayesian course with examples in R and Stan* (Second). Chapman; Hall/CRC.

Nicenboim, B., Schad, D., & Vasishth, S. (2021). An introduction to Bayesian data analysis for cognitive science. *Under contract with Chapman and Hall/CRC statistics in the social and behavioral sciences series*.

Vehtari, A., Gelman, A., Simpson, D., Carpenter, B., & Bürkner, P.-C. (2021). Rank-normalization, folding, and localization: An improved $\hat{R}$ for assessing convergence of mcmc (with discussion). *Bayesian analysis*, *16*(2), 667–718.
