# Supplementary material for: A causal framework for the drivers of animal social network structure
Source: PLoS Comput Biol. 2025 Sep 8;21(9):e1013370. doi: 10.1371/journal.pcbi.1013370 (PMC12459825; doi:10.1371/journal.pcbi.1013370)
Supplement: Attachment — Submitted filename: Response to Reviewers.pdf [file pcbi.1013370.s002.pdf]

Response to reviewers

A causal framework for the drivers of animal social network structure

Ben Kawam[†], Julia Ostner, Richard McElreath, Oliver Schülke[‡], Daniel Redhead[‡]

[†]Corresponding author: *Ben Kawam*, bkawam@dpz.eu.
[‡] These authors contributed equally to this work.

We would like to sincerely thank the two reviewers for engaging thoroughly with the manuscript, for kindly expressing their enthusiasm, and for sharing their detailed suggestions with us. We agreed with almost all of their comments, and have changed the text accordingly. In the few cases where our views contrasted with the reviewers', we detail our arguments below and highlight the changes that have been made, if any.

Response to Reviewer 1

> *My only concern is that someone already familiar with Bayesian inference of hierarchical models, but less so with causal models, might wonder what is the added value of incorporating a formal causal framework for such problems. [...]*

We thank the reviewer for raising this point about the added value of causal inference methods compared to causally ambiguous probabilistic modelling. We start by addressing the specific questions one by one, and then, turn to our conclusions.

> *[...] For example, the difference between the rank-stratified model of Eq. 4.2.3 and its variant without stratification might just be interpreted as the model being misspecified or not. [...]*

In our view, causal inference methods provide researchers with formal tools to reason about *types* of misspecification. The DAG/SCM framework distinguishes, on the one hand, variables that need to be conditioned on to identify an estimand (*e.g.*, confounds): a fatal kind of misspecification; and on the other hand, those variables that are not required for identification (*e.g.*, "competing causes", or "risk-factors"): a more benign type of misspecification (Cinelli et al., 2019). Such insights are particularly valuable to assess the plausibility of empirical causal estimates, as all of the causes of the outcome of interest won't, in most cases, be observed—yet, it is crucial to know whether excluding these unobserved variables is likely to create bias, and therefore, threaten any conclusions.

Furthermore, a causal DAG informs researchers about what variables must be excluded from a model (*i.e.*, what variables must not be conditioned on). This is true of colliders and mediators. Admittedly, total causal effects can still be computed from a model that includes a mediator in principle—yet, if a DAG tells us that we can

exclude it, we might benefit from doing so if our domain expertise is insufficient to confidently specify the functional form of its effect.

In our experience, the process of representing the data-generating process as a DAG also helps researchers reason about selection biases systematically (often, as a form of collider bias). We believe, together with others across Ecology (*e.g.*, Franks et al., 2025), that this issue is strongly underappreciated.

Although we do not have the space to address all of those issues specifically in the manuscript, it is clear to us that providing empiricists with the tools to do so is important—if only because it provides them with a basic toolkit to explore the relevant literature.

> *[...] In other words, following the workflow of Box 1 (page 39), why not go from step 1 directly to step 4 with a statistical generative model? The answer is presumably that a statistical model does not necessarily encode the causal assumptions unambiguously. But in simple examples (like many considered in the manuscript) they are fairly evident from the generative model. I believe this could be expanded more.*

We agree with the reviewer supposition—that causal inference methods disambiguate causal and non-causal concepts. It is important to note that most researchers in animal social network analysis have not received any formal training in probability theory and/or applied statistics. As a result, they tend to treat statistical models as black-boxes that can compute an answer to their question without going through the process of formally defining the "question", nor of understanding how the "answer" is computed.

We think that causal inference methods allow empiricists to, first of all, express their question unambiguously: such methods cannot be bypassed by jumping from step 1 to step 4, for the simple reason that step 1 is already fundamentally causal. Causal quantities (*i.e.*, interventional or counterfactual queries) cannot be expressed by conditional probability statements, or by statistical models alone (Pearl, 2001, 2009).

Second, while empiricists do not generally have an advanced formal training in quantitative methods, they tend to have substantial domain-specific expertise about the causal processes that generate their data. This intuition is fundamentally causal. Thus, we see DAGs and SCMs as a promising set of tools to formalise such expertise, thereby connecting it to statistical estimators transparently.

Reviewer 1 first inquired about the added-value of causal inference methods compared to an explicitly generative yet implicitly causal approach. Given the arguments presented above, we conclude that causal inference methods are indeed necessary for the field's inferential goals. We fear that comparing our framework to an approach that is equally unknown to many of our readers could lead to more confusion than clarification.

We nonetheless read through the manuscript with reviewer 1's concerns in mind, an decided to add a sentence to emphasise that ignoring the open backdoor path—a specific kind of misspecification—would render the estimand unidentifiable (lines 776-778):

> "If we were to regress $m_{[a,b]}$ on $Re_{|a,b|}$ to estimate our estimand without stratifying by rank (as we did in simulation study 3), our effect would be biased by the backdoor path. Note that this would happen even with an infinitely large data set: the causal graph informs us that, because of the backdoor path, our effect simply *cannot* be estimated by looking at the raw association between $Re_{|a,b|}$ and $m_{[a,b]}$."

> *When referring to the literature of Bayesian network reconstruction, the seminal work of Butts should be mentioned:* ref. 1, then, later: *Furthermore, there's a large volume of work on Bayesian network inference using structured priors that is immediately relevant and should also be mentioned, e.g.,* ref. 2, ref. 3, ref. 4.

We added these references—together with (Karrer & Newman, 2011)—to the part of introduction where we mention latent parameter Bayesian models. See lines 96-98.

> *In page 13, line 322, the authors mention that causal effects on count data are "unavoidably" multiplicative, instead of additive. However the model formulation of Eq. 1.1.4 is a choice... It could have been linear, provided γ, ρ, and τ were restricted to non-negative values (also a valid modeling choice).*

Thanks for bringing this nuance. We changed the text accordingly (line 355):

> "We will come back to this issue, because it is a fundamental and unavoidable aspect of modelling behavioural count data with a Generalised Linear Model: causal effects are not simple and additive on the outcome scale, even though we model them as additive on the link scale."

> *With respect to Eqs. 1.1.4 and 1.2.2 and so on, the authors should discuss a potential identifiability problem with the values γ, ρ, and τ and G, R, T, and so on, since it's possible to redistribute their values while keeping their sum fixed, which would preserve the data likelihood. This symmetry is "broken" by the priors on each parameter, but the non-identifiably will still manifest itself in an (artificial) dispersion in the posterior distribution. In some cases like in the degree-corrected SBM this is resolved by normalizing the node parameters (without loss of generality) but this requires a change in the priors.*

We agree that additive models involving varying-effects can suffer from identifiability issues if unconstrained. However, the models presented in the manuscript have no such issues. This is the case because, first, the individual-level parameters G and R are shared across dyads: each $G_{[a]}$ and $R_{[a]}$ appears in $N-1$ linear models, which constrains their values. $T_{[a,b]}$ are the only parameters that appear only once across all linear models. Second, we specified a zero-centred MVNormal prior (eq. 1.2.3) for G, R, and T, effectively defining them as *offsets* from the global intercept D. Together, these modelling specifications ensure that G, R, and T are identifiable, in that they can be uniquely estimated. This theoretical conclusion is confirmed by our simulation results (*e.g.*, Fig. 3). We added a sentence to clarify this issue on line 453-454.

"The distribution, in this case, has two dimensions: G and R. Its mean of $\{0,0\}$ implies that $G_{[a]}$ and $R_{[a]}$ are *deviations* from the average interaction rate in the network, D. Centring the distribution on $\{0,0\}$ also ensures that G and R can be uniquely estimated from the data."

Response to Reviewer 2

My more general comment is, that it would be valuable either in the Introduction section(s) and/or Discussion to highlight how this framework applies or does not apply to other kinds of data common in animal social network analysis. I'm thinking explicitly of non-directional data such as association/gambit-of-the-group data. Both of which are commonly collected and analysed (including in many of the references in the first few sections). The framework presented here is explicitly designed for directional data. I assume the framework could be adapted to deal with non-directional data, but in it's current form the framework is designed for directional data. I don't think it needs to be extended to this kind of data here (there is plenty in this manuscript already!)- but an explicit acknowledgement of this might help avoid confusion, and guide readers to the right framework and tools for the data/questions they have.

We agree it is an important point. We added a paragraph on the topic (see lines 1024-1034) under a new sub-section of the Discussion called Practical application:

"Finally, although the framework presented in Box 1 applies to empirical research across animal social network analysis, the modelling details may require substantial changes—this is notably the case when modelling association data, as measured by spatio-temporal co-occurrences. Association data are not truly dyadic, and much of the observed variation is often unrelated to the underlying social relationships (Carter et al., 2015). Thus, we believe that the models presented in this manuscript may often be poor approximations of the processes generating them. The literature on *hypergraph models* (Battiston et al., 2020; Chodrow & Mellor, 2020; Contisciani et al., 2022; Torres et al., 2021) represents a promising avenue for the analysis of association data, though much work is still needed for accessible and principled causal inference in this context."

Line 17 "M.J. Silk" There are a few points through the manuscript (e.g. line 26 in this paragraph as well) where the references seem to add initials or first names to some authors. Could be worth a careful read through to check.

Thank you for spotting this. We adjusted our reference management settings to ensure only surnames appear in-text, even when authors share the same surname.

Line 23 It might be worth acknowledging that some studies are not interested in the first level of construct e.g. studies of disease spread the realised rate of some behaviour is more important than the social bonds it represents.

We agree. We changed the text accordingly (lines 30-35):

> "In the second level of abstraction, the network edges correspond to one type of quantifiable social interaction. These interactions are generally used as a proxy for the theoretical construct of interest (Carter et al., 2015; Whitehead, 2008)—though it can be the target network in some cases, *e.g.*, when studying disease spread. Hereafter, we refer to this level as the *interaction network* (Figure 1), and assume that it is used as a proxy for a latent construct of interest."

> *Line 81 they also sometimes discard individuals with insufficient data collected...*

This is indeed a serious concern. When we started writing-up the manuscript, we initially wanted to discuss sample selection from the (broader) perspective of collider bias—an issue so important it surely deserves a manuscript on its own. As our manuscript grew, we decided against including this discussion, as we felt the text's length would already be prohibitive to many. To keep our arguments as streamlined as possible, we prefer not to open this pandora's box.

> *Line 82-83 Though attempts have been made, perhaps most influentially: Whitehead, Hal. 'Precision and Power in the Analysis of Social Structure Using Associations'. Animal Behaviour 75 (2008): 1093–99.*

We added the reference in question. See lines 83-84.

> *Line 257-258 It would help DAG amateurs (such as myself) to link "casual" and "non-causal" paths here to the direction of the arrows in the DAG. My reading is the casual is with the arrows, non-casual involves going backwards along an arrow.* Then, *Line 262-263 Similarly I read directed here as "with the arrows".*

We added a few sentences to hopefully clarify these concepts (see lines 263-270):

> "A *path* is a sequence of two or more adjacent nodes (*i.e.*, nodes directly connected by an arrow). For example, consider:
>
> $$X \to Z \to Y.$$
>
> Here, X transmits a causal effect to Y, through a *mediator*, Z. This specific structure (*i.e.*, a path involving three nodes connected by two arrows pointing in the same direction) is referred to as a *chain*. Chains are a type of *causal* paths, as the arrows connecting its two ending points—here, X and Y—go in the same direction. Paths can also be *non-causal*, as in:
>
> $$X \leftarrow Z \to Y.$$
>
> This particular path, where a variable Z is a common cause of two other variables X and Y, is called a *fork*. It is non-causal because one of the arrows between X and Y goes "backwards"."

> *Line 339-341 Might be worth a reminder of what the authors mean about "aggregated indices" could be as simple as just adding an "(e.g. SRI, DSI etc)".*

We changed the text accordingly (line 373):

> "This stands in contrast to aggregated indices of sociality (*e.g.*, Simple Ratio Index) which produce point estimates of rates, without properly characterizing uncertainty, before decomposing them as functions of explanatory variables."

> *Footnote 5. The term "gregariousness" is the word I associate with popularity in social network analyses.*

To comply with PLOS Computational Biology's formatting requirements, we deleted our footnotes, or integrated them in the text. We decided to delete footnote 5, as it discusses the Social Relations Model's terminology in other fields, which is not immediately relevant to behavioural ecologists.

> *Line 515-523 Here you refer to the models as "well-specified" and "non-adjusted" but in the figure it is "adjusted" and "non-adjusted"*

We changed "well-specified" to "adjusted" (line 583).

> *Line 524 If this is intended to be a general introduction a brief unpacking of the word "conditioned" here might help those unfamiliar with the DAG language.*

We added a non-technical definition, higher up in the text, where we use the term for the first time (line 445):

> "Intuitively, conditioning on a variable amounts to saying "once we know its value". It is equivalent to "stratifying by" or "controlling for" it. In the context of a regression model, for example, adding covariates is a form of conditioning."

We also changed the text so as to remind readers of the definition (line 590):

> "Hence, s_G and s_R are larger than 0.5 if we don't stratify by $X_{[a]}$ (right panel), but are equal to 0.5 once we do condition on—or "control for"—it (left panel)."

> *Line 556-561 Why do we expect Sampling effort to effect tau but not rho and gamma? I think I have an answer but it might help readers to have it stated explicitly.*

We expanded on this point (lines 627-631):

> "For the dyad (a, b) to be observed, one has to observe either a or b; thus, the sampling effort for each individual, $S_{[a]}$, in combination with the sampling effort for its partner, $S_{[b]}$, deterministically cause the dyad-level sampling effort, $S_{[a,b]}$—quantifying how long either a or b was observed for—, which in turns affects $m_{[a,b]}$ through $\tau_{[a,b]}$ (for a discussion on deterministic arrows, see Berrie et al., 2022; Koller & Friedman, 2009)."

> *Just below equation 3.2.3 (the line numbers seem to pause for a while here?). Would it be correct or useful to describe T-hat as "realised social bond" or something like it?*

We prefer not to give names to these statistical parameters, because (i) they are defined differently across statistical models, and (ii) their biological interpretation further differs depending on the underlying causal model.

> *Line 655 This line made me expect to see rank modelled as linked to rho- i.e. high ranked individuals are more popular than low ranking individuals. It would be interesting to hear why this was not the case.*

In our SCM, higher-ranked females are modelled as being more popular than lower-ranked ones. We encoded this effect such that the relative popularity of dominant individuals is higher for lower-ranked than for similarly-ranked individuals. We did so (i) because we believe it plausibly captures the underlying mechanism, (ii) to emphasise that dominance hierarchies work through dominance *relationships* between individuals, and (iii) as overall our model aligns with verbal descriptions of how rank influences social interactions (*e.g.*, Hinde, 1983).

We appreciate this comment, as it highlights how translating verbal theory as a set of causal assumptions can bring out potentially important points of disagreement that would likely have remained implicit without formalisation. In this case, specifying the effect of rank through ρ would have been an perfectly valid alternative. However, it would not have changed the main conclusions of the section. Moreover, since most functions used in the manuscript have simple alternatives—as discussed at the end of section 2.1.3—we opted not to elaborate on this particular modelling choice in the text, to avoid unnecessary length.

> *Line 666 Define/remind what an "open path" means*

We added a definition close to lines 595:

> "On a DAG, a path is open if association flows through its components—*i.e.*, two variables connected by an open path are statistically associated. On the other hand, a path is closed when the flow of association among its components is blocked."

> *Line 758 This is an important point and I worry that the word "interacts" might muddy the water, given that a lot of people have an explicit idea of what an interaction (which is not what is being discussed here).*

A statistical interaction occurs when the effect of a variable X on an outcome Y depends on the level of a third variable Z. This definition includes models where an explicit interaction term is included in a linear model—such as those familiar to researchers in the field—*e.g.*,

$$Y = f(\beta_1 X + \beta_2 Z + \beta_3 XZ),$$

but also cases where no interaction term is included in the linear predictor, *e.g.*,

$$Y = f(\beta_1 X + \beta_2 Z),$$

so long as the link function f is non-linear (*e.g.*, exponential, inverse-logit). That is, even without a product term, interactions arise purely because of f. We added the following sentence to clarify this issue (lines 887-889):

> "Generalised linear models (GLM), like statistical model 4, are built-in interaction devices. Contrary to common beliefs, the effect of one parameter on the outcome typically depends on (*i.e.* interacts with) the value of the other predictors in GLMs, even in the absence of an interaction term (Rohrer & Arslan, 2021; Spake et al., 2023)."

P36 above the first psi equation (line numbers paused again). An explicit pointer back to equations 4.2.2 and 4.2.3 might be helpful, I found myself flicking back.

We solved the issue related to line numbers and added a pointer to these equations (lines 904-905).

Line 764 "middle contrast" = C2 ?

Yes! We changed "middle contrast" to C_2 (line 936).

Line 860 I don't quite follow what you mean by:"that is, the list branches out".

We expanded slightly on that (line 1013):

> "In this case, it can be useful to run several analyses in parallel and to compare their outputs—that is, the list of Box 1 bifurcates into a forking paths."

References

Battiston, F., Cencetti, G., Iacopini, I., Latora, V., Lucas, M., Patania, A., Young, J.-G., & Petri, G. (2020). Networks beyond pairwise interactions: Structure and dynamics. *Physics reports*, *874*, 1–92.

Berrie, L., Arnold, K. F., Tomova, G. D., Gilthorpe, M. S., & Tennant, P. W. (2022). Depicting deterministic variables within directed acyclic graphs (DAGs): An aid for identifying and interpreting causal effects involving tautological associations, compositional data, and composite variables. *arXiv preprint*.

Carter, A. J., Lee, A. E., & Marshall, H. H. (2015). Research questions should drive edge definitions in social network studies. *Animal Behaviour*, *104*, E7–E11.

Chodrow, P., & Mellor, A. (2020). Annotated hypergraphs: Models and applications. *Applied network science*, *5*(1), 9.

Cinelli, C., Kumor, D., Chen, B., Pearl, J., & Bareinboim, E. (2019). Sensitivity analysis of linear structural causal models. *International conference on machine learning*, 1252–1261.

Contisciani, M., Battiston, F., & De Bacco, C. (2022). Inference of hyperedges and overlapping communities in hypergraphs. *Nature communications*, *13*(1), 7229.

Franks, D. W., Ruxton, G. D., & Sherratt, T. (2025). Ecology needs a causal overhaul. *Biological Reviews*.

Hinde, R. A. (1983). *Primate social relationships: An integrated approach*. Blackwell Science Ltd.

Karrer, B., & Newman, M. E. (2011). Stochastic blockmodels and community structure in networks. *Physical Review E—Statistical, Nonlinear, and Soft Matter Physics*, *83*(1), 016107.

Koller, D., & Friedman, N. (2009). *Probabilistic graphical models: Principles and techniques*. MIT press.

Pearl, J. (2001). Bayesianism and causality, or, why I am only a half-Bayesian. In *Foundations of Bayesianism* (pp. 19–36). Springer.

Pearl, J. (2009). *Causality*. Cambridge university press.

Rohrer, J. M., & Arslan, R. C. (2021). Precise answers to vague questions: Issues with interactions. *Advances in Methods and Practices in Psychological Science*, *4*(2), 25152459211007368.

Spake, R., Bowler, D. E., Callaghan, C. T., Blowes, S. A., Doncaster, C. P., Antao, L. H., Nakagawa, S., McElreath, R., & Chase, J. M. (2023). Understanding 'it depends' in ecology: A guide to hypothesising, visualising and interpreting statistical interactions. *Biological Reviews*.

Torres, L., Blevins, A. S., Bassett, D., & Eliassi-Rad, T. (2021). The why, how, and when of representations for complex systems. *SIAM Review*, *63*(3), 435–485.

Whitehead, H. (2008). *Analyzing animal societies: Quantitative methods for vertebrate social analysis*. University of Chicago Press.
